# Natural Products from the Lithistida: A Review of the Literature since 2000

**DOI:** 10.3390/md9122643

**Published:** 2011-12-15

**Authors:** Priscilla L. Winder, Shirley A. Pomponi, Amy E. Wright

**Affiliations:** Harbor Branch Oceanographic Institution at Florida Atlantic University, Center for Marine Biomedical and Biotechnology Research, 5600 US 1 North, Fort Pierce, FL 34946, USA; Email: pwinder@hboi.fau.edu (P.L.W.); spomponi@hboi.fau.edu (S.A.P.)

**Keywords:** Lithistida, lithistid, *Theonella*, desmas, natural product

## Abstract

Lithistid sponges are known to produce a diverse array of compounds ranging from polyketides, cyclic and linear peptides, alkaloids, pigments, lipids, and sterols. A majority of these structurally complex compounds have very potent and interesting biological activities. It has been a decade since a thorough review has been published that summarizes the literature on the natural products reported from this amazing sponge order. This review provides an update on the current taxonomic classification of the Lithistida, describes structures and biological activities of 131 new natural products, and discusses highlights from the total syntheses of 16 compounds from marine sponges of the Order Lithistida providing a compilation of the literature since the last review published in 2002.

## 1. Introduction

**Figure 1:**
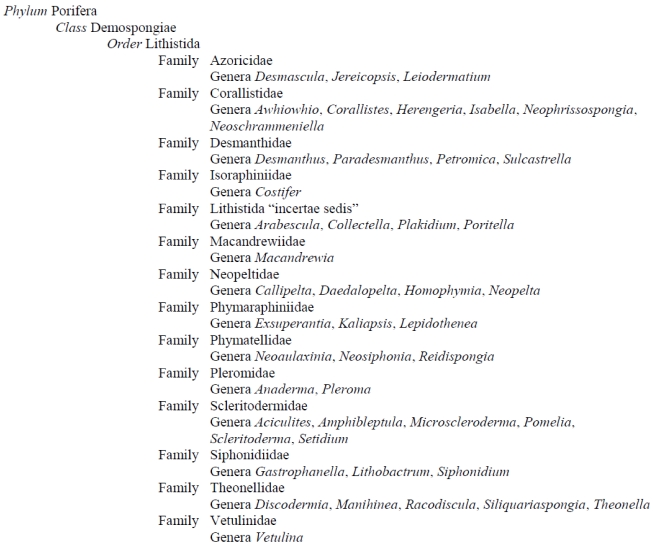
Current classification of lithistid sponges [3].

The Order Lithistida is a polyphyletic assemblage of sponges grouped together based on interlocking siliceous spicules called desmas that make up their skeleton [1,2]. The degree to which the desmas interlock result in lithistid sponges having a firm or rock-hard consistency [1,2]. Many lithistid families and genera have skeletal characteristics that suggest a closer phylogenetic relationship with other sponge taxa. For example, the family Corallistidae is also characterized by the presence of microscleres (amphiasters) that are similar to those found in the Order Astrophorida, Family Pachastrellidae. For many lithistid families and genera, however, skeletal similarities are not as obvious, and until other characters (e.g., molecular data) can be evaluated and analyzed, these desma-bearing sponges will continue to be grouped in the Order Lithistida. A summary of the current taxonomic classification is shown in Figure 1.

Lithistid sponges occur world-wide in both shallow and deep water environments [4]. They are known to produce over 300 different interesting and diverse compounds comprising of polyketides, cyclic and linear peptides, alkaloids, pigments, lipids, and sterols [1,5,6]. Some of their compound diversity has been attributed to the symbiotic microorganisms that reside within the sponge [1,7,8,9]. There have been a few excellent reviews published previously which have highlighted the compounds isolated from lithistid sponges through the year 2000 [1,5,6,10]. A recent review was published that highlighted a subset of bioactive lithistid compounds along with their mechanisms of action [10]. Since the last full review published in 2002, 131 compounds have been reported and substantial success has been achieved in the synthesis of compounds reported from the Lithistida. This review provides an update on the isolation of compounds reported from marine sponges of the Order Lithistida and highlights some of the total synthetic efforts reported since the last review published in 2002 [5].

## 2. Cyclic Peptides

The literature has been dominated by isolations of cyclic peptides from different species within the genus *Theonella* [11]. Since 2000, 51 new cyclic peptides have been reported from five different genera of lithistid sponges [11]. 

**Figure 2:**
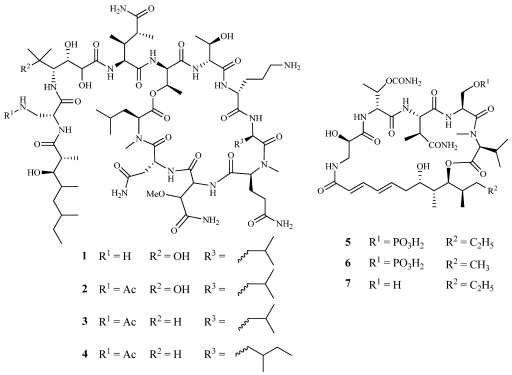
Theopapuamide A (**1**) was isolated from *Theonella swinhoei* and theopapuamides B–D (**2**–**4**) and celebesides A–C (**5**–**7**) were isolated from *Siliquariaspongia mirabilis*.

Theopapuamide A (**1**) is a cytotoxic undecapeptide isolated from *Theonella swinhoei* collected off Milne Bay, Papua New Guinea (Figure 2) [12]. It is the first natural peptide containing *β*-methoxyasparagine and 4-amino-5-methyl-2,3,5-trihydroxyhexanoic acid residues. It was tested in the CEM-TART (T-cells that express both HIV-1 tat and rev) and HCT-116 colorectal carcinoma cell lines with IC_50_ values of 0.5 and 0.9 µM, respectively. In 2009, theopapuamide A (**1**) was reported along with six new cyclic peptides, theopapuamides B–D (**2**–**4**) and celebesides A–C (**5**–**7**), from an extract of *Siliquariaspongia mirabilis* collected off Sulawesi Island, Indonesia (Figure 2) [13]. Compounds **2**, **3**, **5**, and **7** were tested against HCT-116 cells giving IC_50_ values of 2.5, 1.3, 9.9, and >31 µM, respectively. The ability to inhibit HIV-1 entry was also evaluated for **2**, **5** and **7** with IC_50_ values of 0.5, 2.1, and >62 µM. Interestingly for celebesides A and C (**5**,**7**), in both biological assays, loss of activity correlated with the loss of the phosphate group. **1**–**3** were evaluated for their ability to inhibit the growth of both wild type and amphotericin B-resistant strains of *Candida albicans*. **1** inhibited the growth of both strains with zones of inhibition of 8 mm at 1 µg/disk while **2** and **3** displayed zones of 10 mm against both strains at 5 µg/disk.

**Figure 3:**
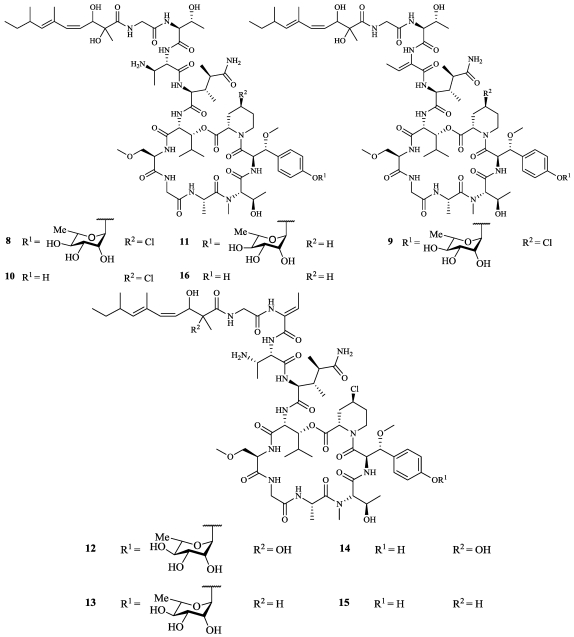
Mirabamides A–D (**8**–**11**) were isolated from *Siliquariaspongia mirabilis* while mirabamides E–H (**12**–**15**) were isolated from the Astrophorid sponge *Stelleta calvosa*. Papuamide A (**16**) is a related compound.

A specimen of *Siliquariaspongia mirabilis* collected off Nama Island, southeast of Chuuk Lagoon, in the Federated States of Micronesia yielded the mirabamides A–D (**8**–**11**), which are potent inhibitors of HIV-1 entry (Figure 3) [14]. **8**–**11** were tested in an HIV-1 neutralization assay which tests a compound’s ability to neutralize the biological effects of the HIV-1 virus on the TZM-bl cell line and used two different viral strains: HXB2 (T-cell tropic) and SF162 (macrophage-tropic). Against the HXB2 virus, IC_50_ values for **8**, **10**, and **11** were 140, 140, and 189 nM and against the SF162 virus, **8**, **10**, and **11** were slightly less active with IC_50_ values of 0.40, 1.01, and 1.31 µM. **8**–**11** were also tested in an HIV-1 fusion assay that tests the ability of a compound to inhibit envelope-mediated cell fusion against the LAV (T-cell tropic) viral strain. In the fusion assay, IC_50_ values for **8**, **10**, and **11** were 0.041, 1.3, and 3.9 µM. **9** did not show inhibition in any of the antiviral assays under the conditions tested. **8**, **10**, and **11** were tested against the neutralization assay host cell line, TZM-bl showing IC_50_ values of 1.8, 2.2, and 3.9 µM, respectively. **9** was tested against the HCT-116 cell line with an IC_50_ value of 2.22 µM. Mirabamides E–H (**12**–**15**) along with **10** were recently isolated from a sponge, *Stelleta clavosa*, from the Order Astrophorida collected in the Torres Strait, Queensland, Australia (Figure 3) [15]. **10** and **12**–**15** were tested in the HIV-1 neutralization assay using the viral strain YU2-V3 with IC_50_ values of 123, 121, 62, 68, and 42 nM, respectively. Mirabamides A–D (**8**–**11**) are very similar in structure to papuamide A (**16**) but differ by the presence of the novel 4-chlorohomoproline and *β*-methoxytyrosine 4′-*Ο*-*α-*L-rhamnopyranoside residues (Figure 3) [16]. **9**, which had no biological activity, contains one additional alteration in which the 2,3-diaminobutanoic acid residue is replaced with 2-amino-2-butenoic acid. Mirabamides E–H (**12**–**15**) differ from **8**–**11** by the replacement of the threonine residue with 2-amino-2-butenoic acid. Papuamide A (**16**) was also tested in the fusion assay and against the HCT-116 cell line with IC_50_ values of 73 nM and 3.5 µM. The anti-HIV activity of **16** has recently been determined to be through a membrane targeting mechanism in which the hydrophobic tail of the molecule inserts into the viral membrane and the tyrosine residue interacts with cholesterol [17].

**Figure 4:**
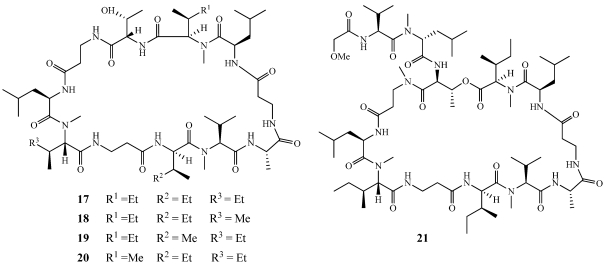
Barangamides A–D (**17**–**20**) and theonellapeptolide IIe (**21**) were isolated from an Indonesian collection of *Theonella swinhoei*.

Barangamides A–D (**17**–**20**) were isolated from a specimen of *Theonella swinhoei* collected at Baranglompo Island, Indonesia along with theonellapeptolide IIe (**21**) and a series of previously reported theonellapeptolides from the I and II series (Figure 4) [18,19]. The amino acid sequence of **17** is the same as the cyclic portion of the theonellapeptolide II series but **17** lacks the amino acid side chain. In the barangamide series, macrocyclization occurs through peptide bond formation of the amine of the threonine rather than through lactonization of the hydroxyl group of threonine as found in the theonellapeptolide series. Because theonellapeptolides Ia-Ie were previously known to be moderately cytotoxic against the L1210 mouse lymphocytic leukemia cell line, **17** was tested but no cytotoxicity was observed at concentrations up to 9.4 μM [18,19,20]. Cyclic undecapeptides share structural similarities with the cyclosporins which are used as immunosuppresants after organ transplants [21]. The immunomodulatory activity of the known theonellapeptolides Ia, Id, and IId as well as **17** were analyzed in the mixed lymphocyte reaction (MLR) assay [20]. Barangamide A (**17**) showed no activity even at the highest concentration of 94 μM while theonellapeptolide IId showed the strongest immunosuppressive activity. 

**Figure 5:**
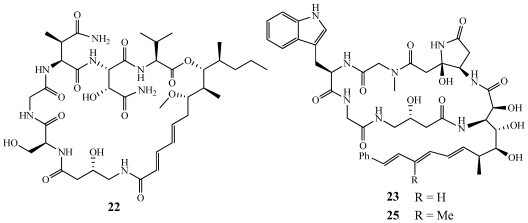
Nagahamide A (**22**) was isolated from a Japanese collection of *Theonella swinhoei* and microsclerodermins F (**23**) and H (**25**) were isolated from *Microscleroderma* sp.

Nagahamide A (**22**) was isolated from *Theonella swinhoei* collected near Nagahama, Kamikoshiki-jima Island, Japan (Figure 5) [22]. It was purified using bioassay-guided fractionation following anti-fungal activity. Once purified, **22** showed weak antibacterial activity against *Escherichia coli* and *Staphylococcus aureus* with 7 mm zones of inhibition when tested at 50 μg/disk but no antifungal activity was observed against *Saccharomyces cerevisiae* or *Mortierella ramanniana* at the same dose. Of the seven residues in **22**, two were unusual: 8,10-dimethyl-9-hydroxy-7-methoxytrideca-2,4-dienoic acid (DHMDA) and 4-amino-3-hydroxybutanoic acid (or *gamma*-amino-*beta*-hydroxybutyric acid, GABOB), which is also found in the cytotoxic, anti-fungal microsclerodermins [23,24]. 

**Figure 6:**
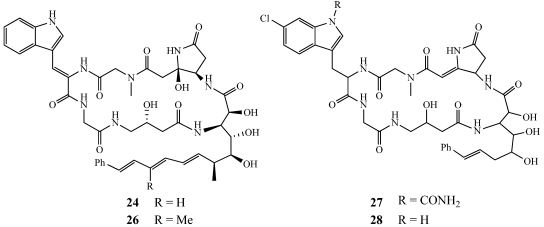
Microsclerodermin G (**24**) and I (**26**) were isolated from a deep-water collection of *Microscleroderma* sp. Dehydromicrosclerodermins C–D (**27**–**28**) were isolated from an Okinawan collection of *Theonella cupola*.

Microsclerodermins A–E were discussed in earlier reviews yet further studies on *Microscleroderma* sp. from a deep-water specimen collected off Short Dropoff, Koror, Palau afforded microsclerodermins F–I (**23**–**26**) (Figure 5 and Figure 6) [25]. **23**–**26** showed very similar cytotoxicity against the HCT-116 cell line with IC_50_ values of 1.1, 1.2, 2.0, and 2.6 µM, respectively. They were also tested for the ability to inhibit the growth of *C. albicans* using a paper disk diffusion assay with a minimum concentration in which inhibition was observed of 1.5, 3, 12, and 25 mg/disk, respectively. **23**–**26** differ from previously published microsclerodermins by alterations in the *ω*-aromatic 3-amino-2,4,5-trihydroxyacid residue and **24** and **26** also have a modification in the tryptophan moiety. The dehydromicrosclerodermins C–D (**27**–**28**) were isolated as the major constituents from an Okinawan collection of *Theonella cupola* (Figure 6) [26]. 

**Figure 7:**
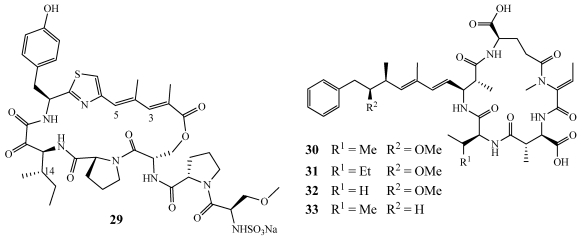
Scleritodermin A (**29**) was isolated from *Scleritoderma nodosum* and isomotuporins A–D (**30**–**33**) were isolated from *Theonella swinhoei*.

From the same family as *Microscleroderma*, specimens of *Scleritoderma nodosum* were collected from the northwest side of Olango Island, Cebu, Philippines and from Milne Bay, Papua New Guinea. These specimens yielded the cytotoxic cyclic peptide, scleritodermin A (**29**) (Figure 7) [27]. **29** was tested against the HCT-116, the HCT-116/VM46 multidrug-resistant colon cancer, the A2780 human ovarian carcinoma, and the SKBR3 breast carcinoma cell lines with IC_50_ values of 1.9, 5.6, 0.940, and 0.670 µM, respectively. Cell cycle analysis in A2780 cells treated with scleritodermin A (**29**) for 24 h at a concentration of 1.3 µM yielded a G2/M block. As a G2/M block is characteristic of compounds that target tubulin, **29** was studied further and found to inhibit GTP-induced tubulin polymerization by 50% at a concentration of 10 µM. **29** caused a 5.5-fold increase in the induction of apoptosis over the control after a 24 h drug exposure at a concentration close to its cytotoxic IC_50_. Since scleritodermin A (**29**) had significant *in vitro* cytotoxicity in human tumor cell lines as well as an *O*-methyl-*N*-sulfoserine, a novel conjugated thiazole moiety, and an R-ketoamide group, its total synthesis was undertaken [28,29]. The initial structure was assigned based upon NOESY data as the 2*Z*, 4*E* configuration for the conjugated thiazole moiety. The structure was revised to the 2*E*, 4*E* configuration following synthesis based upon the observation of the methine protons, CH-3 and CH-5, 1.0 ppm further upfield than in the natural product [28,29]. In addition, a difference was observed for the chemical shifts of the two methyl groups in the keto-Ile moiety revising the 14*R* assignment to a 14*S*-configuration. 

During an LC-MS screening study by the Crews group to predict which phenotypes of *Theonella swinhoei* contain swinholide A or motuporin, they isolated another series of compounds called the isomotuporins A–D (**30**–**33**) (Figure 7) [30,31,32,33,34,35]. The loss of the methoxy group in the 3-amino-9-methoxy-10-phenyl-2,6,8-trimethyldeca-4,6-dienoic acid (ADDA) residue in isomotuporin D (**33**) is the first report of that variation from a natural source. Most importantly, this study provides further assistance for researchers probing for the swinholide or motuporin biosynthetic pathways and also demonstrates that some populations produce either 2*S*-motuporin A or 2*R*-motuporin A (**30**). 

**Figure 8:**
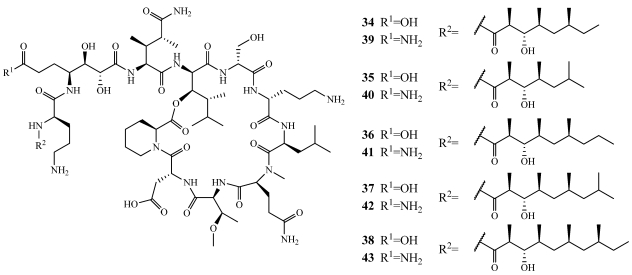
Homophymines A–E (**34**–**38**) and A1–E1 (**39**–**43**) were isolated from a New Caledonian collection of *Homophymia* sp.

Homophymines A–E (**34**–**38**) and A1–E1 (**39**–**43**) are a series of cyclodepsipeptides isolated from *Homophymia* sp. collected from shallow waters off the east coast of New Caledonia (Figure 8) [36,37]. They are similar in structure to the previously published antiviral marine cyclodepsipeptides, callipeltin A, neamphamide A, papuamides, theopapuamides (**1**–**4**), and mirabamides (**8**–**15**) [12,13,14,15,16,38,39]. **39**–**43** differ from **34**–**38** due to an amide moiety in place of the carboxylic acid on the 4-amino-2,3-dihydroxy-1,7-heptandioic acid residue. The anti-viral properties of **34** were tested in an assay with peripheral blood mononuclear cells (PBMC) infected with the III B strain of HIV-1. **34** had cytoprotective properties by inhibiting the production of an infection with an IC_50_ value of 75 nM. Homophymine A (**34**) was cytotoxic against uninfected PBMC cells with an IC_50_ of 1.19 µM but it was almost sixteen times more effective against infected cells. **34**–**43** were evaluated against a panel of cell lines including human cancer and the Vero green monkey kidney cell lines. **34**–**43** exhibited potent cytotoxicity with IC_50_ values ranging from 2 to 100 nM. They were the most potent in the PC3 human prostate adenocarcinoma and the SK-OV3 human ovarian adenocarcinoma cell lines. Further studies were performed on **34**–**43** to determine if they were toxic or antiproliferative. They were found to undergo apoptosis through a caspase independent pathway but were ultimately determined to exert their toxicity through an acute direct and non specific mechanism. 

**Figure 9:**
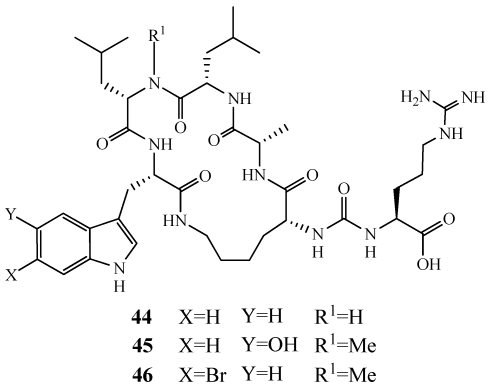
Paltolides A–C (**44**–**46**) were isolated from a specimen of *Theonella swinhoei* collected in deep-water off Palau.

Paltolides A–C (**44**–**46**) were isolated from a deep-water specimen of *Theonella swinhoei* collected off Uchelbeluu Reef in Palau (Figure 9) [40]. These are anabaenopeptin-type compounds structurally similar to patented compounds isolated from an Australian sponge *Melophlus* sp. [41]. Compounds within the anabaenopeptin class contain an *N*-methylated amino acid adjacent to and before the *C*-terminal residue which is cyclized to the ε-amine of the lysine residue. **44** contains a standard leucine residue at this site and is the first report of an anabaenopeptin-type peptide lacking an *N*-methyl group at this site. These compounds are part of a rare subgroup of the anabaenopeptins since they contain a *C*-terminal tryptophan residue linked to the ε-amine of the *N*-terminal lysine residue. The other compounds within this subgroup are known to inhibit carboxypeptidase U. **44** and **45** did not have biological activity in the HIV-1 entry assay or against HCT-116 but their ability to inhibit carboxypeptidase U has not been evaluated. 

**Figure 10:**
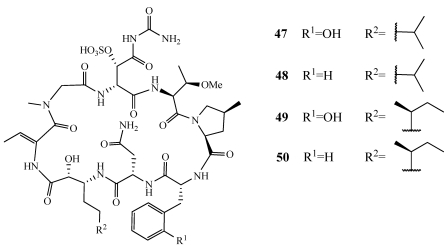
Mutremdamide A (perthamide C, **47**) and perthamides D–F (**48**–**50**) were isolated from various specimens of *Theonella swinhoei*.

Mutremdamide A (**47**) was isolated from a few deep-water specimens of *Theonella swinhoei* subspecies *swinhoei*, *Theonella swinhoei* subspecies *verrucosa*, and *Theonella cupola* collected from Mutremdiu Reef, Palau, at depths of 90–120 m using SCUBA (Figure 10) [42]. Mutremdamide A (**47**) is a sulfated cyclic depsipeptide related to perthamide B isolated previously from an Australian specimen of *Theonella* sp. [43]. **47** differs in three of the eight residues and contains a new *N^δ^*-carbamoyl-*β*-sulfated asparagine residue as well as the rare *o*-tyrosine residue. Initially, **47** was reported as perthamide C isolated alongside perthamide D (**48**) from *Theonella swinhoei* collected from the barrier reef of Vangunu Island, Solomon Islands (Figure 10) [44]. In the original assignment for perthamide C, a *β*-hydroxyasparagine rather than the *N^δ^*-carbamoyl-*β*-sulfated asparagine residue was proposed but its structure has since been revised to that of **47 [45]**. Perthamide D (**48**) contains a phenylalanine in place of the *o*-tyrosine residue in **47**. The anti-inflammatory activity of **47** and **48** was evaluated *in vivo* using the mouse paw edema model [44]. **47** reduced carrageenan-induced paw edema in a dose-dependent manner in both early (0–6 h) and late (24–96 h) phases. **47** and **48** displayed a 60% and 46% reduction of edema at 0.3 mg/kg. Based on current NSAIDs on the market such as naproxen (ED_50_ 40 mg/kg), **47** is nearly 100 times more potent. Perthamides E (**49**) and F (**50**) were isolated from a *Theonella swinhoei* collected on a reef at a depth of 22 m from the western coast of Malaita Island, Solomon Islands (Figure 10) [46]. **47** and **50** have a 3-amino-2-hydroxy-6-methyloctanoic acid (AHMOA) residue in place of the 3-amino-2-hydroxy-6-methylheptanoic acid (AHMHA) residue. Based on the anti-inflammatory activity in the mouse models, **47**–**50** were evaluated for their antipsoriatic effects on TNF-α and IL-8 release using primary human keratinocytes (PHK) cells. Although **48** and **50** were too cytotoxic at concentrations up to 10 µM, **47** showed a dose-dependent response to inhibit the release of both TNFa and IL-8 and **49** significantly inhibited the release of IL-8 [46]. 

**Figure 11:**
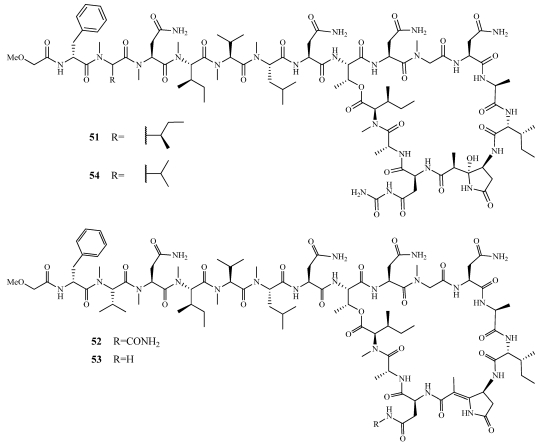
Koshikamide B (**51**) was isolated from *Theonella* sp. and koshikamides F–H (**52**–**54**) were isolated from *Theonella swinhoei*.

The cytotoxic cyclic peptide lactone, koshikamide B (**51**), is the first account of a peptide possessing a carbamoylated asparagine and the new amino acid residue 2-(3-amino-2-hydroxy-5-oxopyrrolidin-2-yl) propionic acid (AHPP) (Figure 11) [47]. It was initially isolated from a *Theonella* sp. collected off Shimokoshiki Island, Kagoshima Prefecture, Japan but was later found in a *Theonella* sp. collected from Palau. It exhibited cytotoxicity against P388 murine leukemia and HCT-116 human colon tumor cell lines with IC_50_ values of 0.22 and 3.7 µM, respectively. Koshikamides F–H (**52**–**54**) are 17-residue depsipeptides containing a 10-residue macrolactone isolated alongside mutremdamide A (**47**) (Figure 10 and Figure 11) [42]. **54** differs from **51** by the substitution of *N*MeIle with *N*MeVal. **52** is structurally similar to **54** and contains a (*Z*)-2-(3-amino-5-oxopyrrolidin-2-ylidene) propanoic acid in place of the AHPP residue. **53** was determined by MS and NMR to be the descarbamoyl derivative of **52**. Compounds **52** and **54** were tested in a single round HIV-1 neutralization assay against the SF162 strain. In the assay, **52** and **54** inhibited entry with IC_50_ values of 2.3 and 5.5 µM, respectively. **54** also had moderate cytotoxicity in the HCT-116 colon cancer cell line with an IC_50_ of 10 µM. **52**–**54** did not inhibit the growth of *Candida albicans*. 

**Figure 12:**
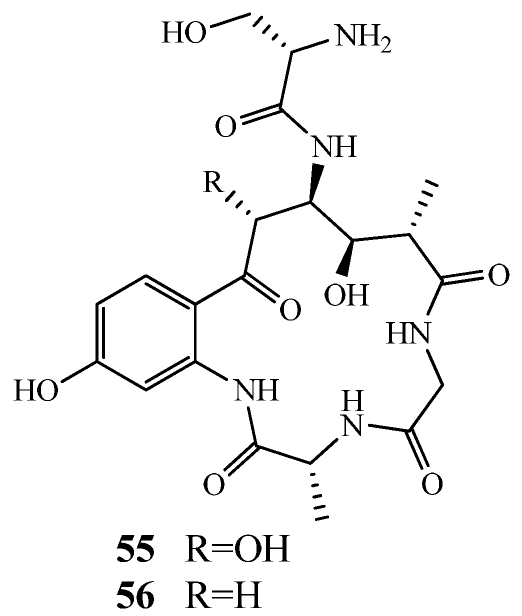
A collection of *Theonella swinhoei* from the Solomon Islands yielded solomonamides A and B (**55**,**56**).

Solomonamides A and B (**55**–**56**) are 4-residue cyclic peptides with a unique 4-amino-6-(2’-amino-4’-hydroxyphenyl)-3,5-dihydroxy-2-methyl-6-oxohexanoic acid residue isolated from the same specimen of *Theonella swinhoei* that contained perthamides C and D (**47**–**48**) (Figure 10 and Figure 12) [48]. **55** displayed a dose-dependent anti-inflammatory response causing nearly a 60% reduction of edema in mice at a dose of 100 μg/kg (ip.). The absolute configuration of **55** was established through extensive work including Marfey’s method, Quantum Mechanical *J* based analysis, and Density Functional Theory (DFT) *J*/^13^C calculations. 

## 3. Linear Peptides

**Figure 13:**
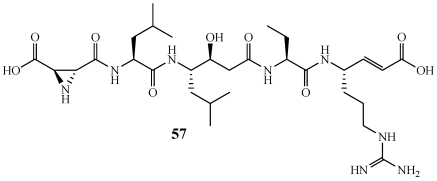
Miraziridine A (**57**) was isolated from a Japanese collection of *Theonella* aff. *mirabilis*.

Miraziridine A (**57**) is a linear five amino acid peptide isolated from a specimen of *Theonella* aff. *mirabilis* collected off the Amami and Tokara Islands, Japan (Figure 13) [49]. It contains the rare aziridine-2,3-dicarboxylic acid residue that has only been reported one other time from a *Streptomyces* sp. and also a vinylogous arginine residue that has never before been reported from a natural source. **57** inhibited the enzymatic activity of cathepsin B with an IC_50_ value of 2.1 µM. The total synthesis of **57** has been completed (discussed later in this review [50,51]).

**Figure 14:**
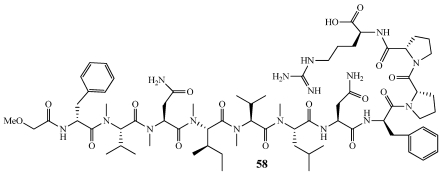
Koshikamide A_2_ (**58**) was isolated from a Japanese collection of *Theonella* sp.

Koshikamide A_2_ (**58**) was isolated from a *Theonella* sp. collected off Shimo-koshiki-jima Island, Kagoshima, Japan (Figure 14) [52]. The structure of **58** was determined to be a close structural homolog of the previously described Koshikamide A_1_ with an additional arginine residue added to the *C*-terminus. **58** was cytotoxic to P388 murine leukemia cell line with an IC_50_ value of 4.6 µM whereas Koshikamide A_1_ was more cytotoxic with an IC_50_ value of 1.7 µM. 

**Figure 15:**
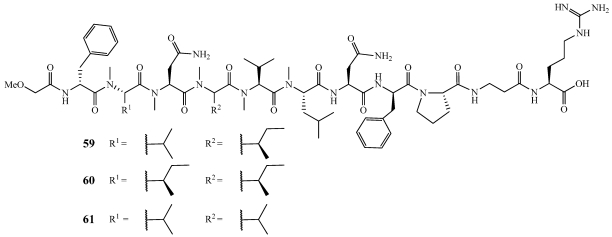
Koshikamides C–E (**59**–**61**) were isolated from various specimens of *Theonella swinhoei* and *T. cupola* from the reefs of Palau.

Koshikamides C–E (**59**–**61**) are linear undecapeptides isolated alongside **47**, and **52**–**54** (Figure 10, Figure 11, and Figure 15) [42]. Their structures were determined by extensive NMR and mass spectrometry. **59** was observed to exist as two stable conformers due to *cis*/*trans* isomerization. They were tested in a single round HIV-1 infectivity assay against a CCR5-using viral envelope but no biological activity was observed. 

**Figure 16:**
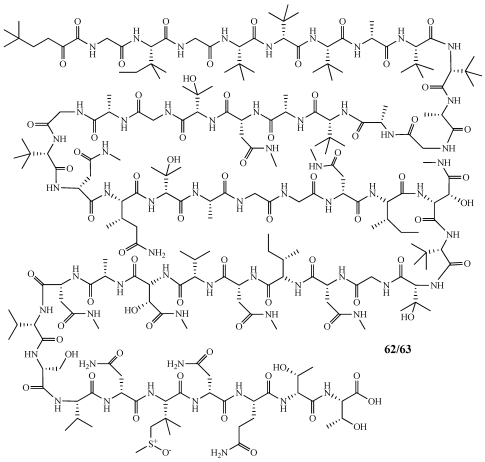
The 48-residue linear peptides, polytheonamides A and B (**62**,**63**), were isolated from a Japanese collection of *Theonella swinhoei*.

Polytheonamides A (**62**) and B (**63**) were isolated from a specimen of *Theonella swinhoei* collected off Hachijo-jima Island, Japan and initially reported in 1994 by the Fusetani group at Tokyo University (Figure 16) [53,54]. During their studies to determine the configuration of **62** and **63**, they realized the initially proposed structure was incorrect and determined the new structure by spectral and chemical methods, relying heavily on 2D NMR experiments [53]. **62** and **63** are 48 amino acid residue polypeptides with numerous unprecedented structural features such as the unusual *N*-terminal blocking group, the first report from a natural source of the 5,5-dimethyl-2-oxoheptanoyl group, the presence of eight rare *t*-Leu residues from a marine source, and the first report of the residue *β*,*β*-dimethylmethionine sulfoxide. **62** and **63** are isomeric at the sulfoxide moiety on the 44th residue. **62** and **63** are the largest non-ribosomal peptides with an alternating D/L stereochemistry throughout the chain seen only once before in gramicidin A, which is a linear 15-residue peptide produced by *Bacillus brevis* [55]. 

## 4. Polyketides and Macrolides

**Figure 17:**
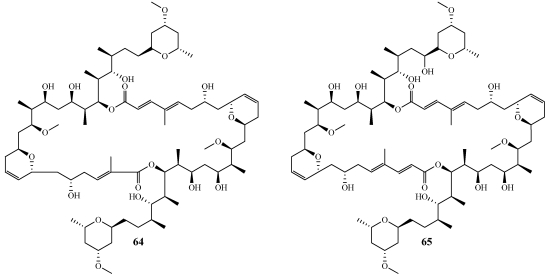
Hurghadolide A (**64**) and swinholide I (**65**) were isolated from an Egyptian collection of *Theonella swinhoei*.

**Figure 18:**
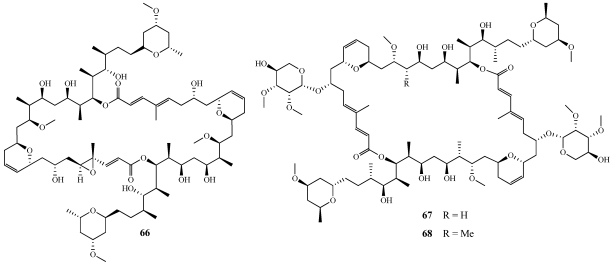
Swinholide J (**66**) was isolated from *Theonella swinhoei* and ankaraholides A and B (**67**,**68**) were isolated from the cyanobacteria, *Geitlerinema* sp.

Hurghadolide A (**64**) and Swinholide I (**65**) were isolated from a specimen of *Theonella swinhoei* collected in Hurghada at the Egyptian Red Sea coast (Figure 17) [56]. Hurghadolide A (**64**) is one acetate unit shorter than **65** and therefore has an unprecedented asymmetric 42-membered dilactone moiety which represents a new macrolide carbon skeleton. Swinholide I (**65**)is similar in structure to swinholide A and is the first swinholide derivative with hydroxylation on the side chain [57]. **64** and **65** showed very potent *in vitro* cytotoxicity against HCT-116 with IC_50_ values of 5.6 and 365 nM, respectively, as well as disruption of the actin cytoskeleton at concentrations of 70 and 7.3 nM, respectively. Also, both compounds were tested for their ability to inhibit the growth of *C. albicans* with MIC values of 31.3 and 62.2 µg/mL, respectively. Swinholide J (**66**) was isolated from a specimen of *Theonella swinhoei* collected on the reef off Vangunu Island, Solomon Islands (Figure 18) [58]. **66** has an unprecedented asymmetric 44-membered dilactone moiety and contains an epoxide functionality in one half of the molecule. **66** and swinholide A showed very potent *in vitro* cytotoxicity against the KB nasopharyngeal epidermoid carcinoma cell line with IC50 values of 6.7 and 1.2 nM, respectively. 

In 2005, the Gerwick group reported the isolation of swinholide A (initially isolated in 1985 from the sponge *Theonella swinhoei*) and two glycosylated derivatives, ankaraholide A (**67**) and B (**68**), for the first time from field collections of two species of cyanobacteria (Figure 18) [7]. Specimens of *Symploca* cf. sp. that produced swinholide A were collected from the Fiji Islands while specimens of *Geitlerinema* sp. collected from Nosy Mitso-ankaraha Island, Madagascar produced **67** and **68**. This discovery as well as the fact that the swinholides are produced by three taxonomically unrelated sponges suggests that symbiotic microorganisms may be the true producers of these metabolites. Since the swinholides cause cytotoxicity by disrupting the actin cytoskeleton, the effects of the added sugar moiety were evaluated. **67** caused cytotoxicity in NCI-H460 human large cell lung cancer, Neuro-2a mouse brain neuroblast, and MDA-MB-435 human melanoma cell lines with IC_50_ values of 119, 262, and 8.9 nM, respectively. In A-10 aortic smooth muscle cells, **68** caused the complete loss of filamentous actin at 30 and 60 nM which is consistent with the disruption of the actin cytoskeleton. 

**Figure 19:**
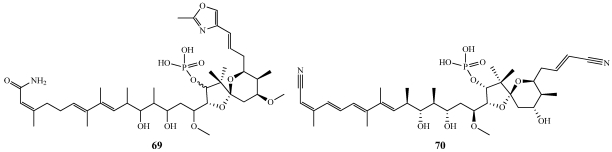
Swinhoeiamide A (**69**) was isolated from a Papua New Guinea collection of *Theonella swinhoei*. Hemicalyculin (**70**) was isolated from a Japanese collection of *Discodermia calyx*.

A new calyculinamide-related congener called swinhoeiamide A (**69**) was isolated from *Theonella swinhoei* collected off the coast of the Karkar Island, Papua New Guinea (Figure 19) [59]. The structure of the new compound was assigned on the basis of 1D and 2D NMR spectroscopy and HRMS data. **69** differs from calyculinamide A by replacement of the complex side chain attached to C-29 in the oxazole ring system with a methyl substituent and **69** is also the first calyculin congener in which the double bond at C-4 and C-5 is hydrogenated [60]. **69** exhibited insecticidal activity toward the neonate larvae of the insect *Spodoptera littoralis* in a chronic feeding bioassay with an effective dose, 50% (ED_50_) value of 2.11 ppm and a median lethal dose (LD_50_) value of 2.98 ppm. **69** was found to inhibit the growth of *C. albicans* and *Aspergillus fumigatus* with MIC values of 1.2 and 1.0 µg/mL, respectively. **69** also exhibited dose-dependent cytotoxicity against various undisclosed cell lines and tissues with IC_50_ values ranging between 20 and 90 ng/mL. Another calyculin A derivative, hemicalyculin A (**70**), was isolated from *Discodermia calyx* collected off Sikine-jima Island, Japan (Figure 19) [61]. The structure of **70** is comprised of just the southern hemisphere of calyculin A and allowed the Fusetani group to pursue structure-activity relationships providing further insight into the binding of calyculin A with protein phosphatases 1 and 2A (PP1 and PP2A) [61,62,63,64]. **70** was tested alongside calyculin A to determine its ability to inhibit PP1 and PP2A with IC_50_ values of 14.2 nM and 1.0 nM *versus* 8.2 nM and 1.0 nM of calyculin A. 

**Figure 20:**
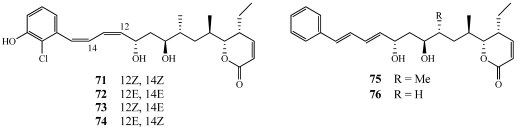
Bitungolides A–F (**71**–**76**) were isolated from an Indonesian collection of *Theonella* cf. *swinhoei*.

Bitungolides A–F (**71**–**76**) were isolated from a specimen of *Theonella* cf. *swinhoei* collected along the Lembeh Strait off Bitung, Sulawesi Island, Indonesia (Figure 20) [65]. The structure of **71** was confirmed by a single-crystal X-ray diffraction study. Bitungolides are structurally similar to pironetin reported previously from a *Streptomyces* sp. [66]. Pironetin shows moderate *in vivo* anti-tumor activity and arrests cells at the M-phase of the cell cycle [67]. **71**–**76** were tested against a number of phosphatases and showed no activity against protein tyrosine phosphatase-S2 (PTP-S2), PP1, or PP2A. **71**–**76** showed weak activity against dual-specificity protein phosphatase vaccinia H1-related (VHR). The total synthesis of bitungolide F has been completed and will be discussed in a later section [68]. 

**Figure 21:**
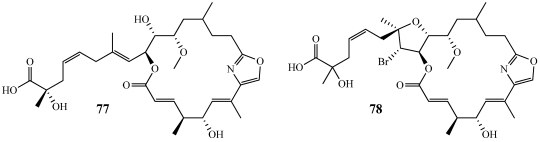
Leiodolides A and B (**77**,**78**) were isolated from a deep-water sponge, *Leiodermatium* sp. collected in Palau.

Leiodolides A (**77**) and B (**78**) were isolated from a new species of the deep-water sponge *Leiodermatium* sp. collected at 240 m near Uchelbeluu Reef in Palau using the manned submersible Deep Worker (Figure 21) [69]. **77** and **78** are the first published compounds from this genus of sponge. The leiodolides represent the first members of a new class of 19-membered ring macrolides and they incorporate several unique functional groups such as a conjugated oxazole ring, a bromine substituent, and an α-hydroxy-α-methyl carboxylic acid side-chain terminus. **77** and **78** were cytotoxic against the HCT-116 cell line with IC_50_ values of 2.5 and 5.6 µM, respectively. In the NCI 60 cell line panel, leiodolide A (**77**) was cytotoxic to the HL-60 leukemia, the NCI-H522 non-small cell lung cancer, and the OVCAR-3 ovarian cancer cell lines with growth inhibition, 50% (GI_50_) values of 0.26, 0.26, and 0.25 µM, respectively. Recently, a total synthesis of the proposed structure for leiodolide B (**78**) was published, but its spectroscopic data did not match those of the authentic sample [70]. This will be discussed in a later section.

**Figure 22:**
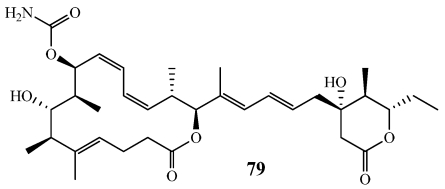
Leiodermatolide (**79**) was isolated from a Floridian collection of the deep-water lithistid, *Leiodermatium* sp.

Leiodermatolide (**79**) was isolated from *Leiodermatium* sp. collected on the Miami Terrace in the Straits of Florida at 401 m using the Johnson-Sea-Link submersible (Figure 22) [71]. It has a 16-membered macrolide ring with a carbamate and a substituted lactone in the side chain. **79** was isolated by bioassay guided fractionation following a Phospho-nucleolin Cytoblot Assay where **79** showed potent inhibition of mitosis. Further studies found that **79** induced a G2/M block in the cell cycle. **79** was tested against the A549 human lung adenocarcinoma, the NCI-ADR-RES human ovarian sarcoma, the P388 murine leukemia, the PANC-1 human pancreatic carcinoma, and the DLD-1 colorectal adenocarcinoma cell lines with very potent IC_50_ values of 3.3, 233, 3.3, 5.0, and 8.3 nM. 

**Figure 23:**
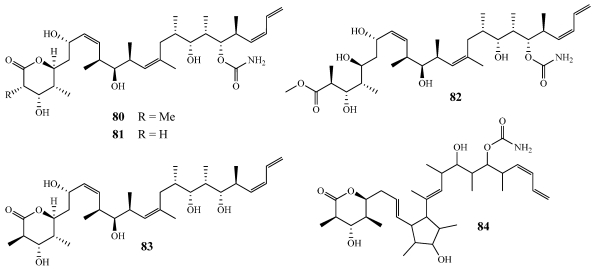
Five new discodermolide analogues reported from *Discodermia* sp.: 2-epidiscodermolide (**80**), 2-desmethyldiscodermolide (**81**), 5-hydroxymethyl- discodermolide (**82**), 19-des-aminocarbonyldiscodermolide (**83**), and 9(13)-cyclodiscodermolide (**84**).

Collections of *Discodermia* sp. obtained using the Johnson-Sea-Link submersible in numerous places throughout the Bahamas yielded five new discodermolide analogues: 2-epidiscodermolide (**80**), 2-desmethyldiscodermolide (**81**), 5-hydroxymethyl- discodermolide (**82**), 19-des-aminocarbonyldiscodermolide (**83**), and 9(13)-cyclodiscodermolide (**84**) (Figure 23) [72]. Based on these five natural analogs as well as numerous synthetic derivatives, information about the structure-activity relationship of discodermolide was obtained. Discodermolide and its naturally occurring analogues **80**–**84** were tested against the P-388 cell line with IC_50_ values of 35, 134, 172, 65.8, 128 and 5043 nM as well as the A-549 cell line with IC_50_ values of 13.5, 67, 120, 74, 74 and 4487 nM [72,73]. This data indicated that alterations in the *δ*-lactone ring have a minor contribution toward the activity but changes at the diene end of the molecule had no significant decrease in activity. Complete loss of activity was observed when alterations were made in the middle section of the molecule. This data was in agreement with earlier findings that the C-7 through C-17 backbone, which adopts a hairpin conformation, is essential to the cytotoxicity of discodermolide [74,75]. 

**Figure 24:**
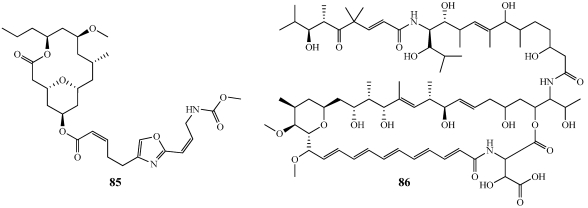
Neopeltolide (**85**) was isolated from a deep-water sponge of the Family Neopeltidae. Mirabalin (**86**), initially published as mirabilin, was isolated from *Siliquariaspongia mirabilis*.

Neopeltolide (**85**) was isolated from two samples of sponge from the Family Neopeltidae collected using the Johnson-Sea-Link submersible at depths of 442 and 433 m off the northwest coast of Jamaica (Figure 24) [76]. **85** is a potent inhibitor of the proliferation of the A-549, NCI-ADR-RES, and P388 cell lines with IC_50_ values of 1.2, 5.1, and 0.56 nM, respectively. **85** also inhibits the growth of *C. albicans* with an MIC of 0.62 µg/mL. **85** is structurally homologous to the potent antiproliferative compound leucascandrolide A and the cytochrome *bc*_1_ complex was found to be the primary cellular target of both compounds [77]. Based on its potent biological activity and similarity to leucascandrolide A, **85** was targeted by numerous synthetic groups to complete its total synthesis and absolute configuration. The relative configuration of **85** was suggested as 11*R*, 13*R* based on coupling constant analysis, 2D-NOESY, and a series of double-pulsed field gradient spin echo (DPFGSE) NOE experiments but once it was synthesized, its configuration was reassigned to 11*S*, 13*S* [78,79]. The total synthesis of **85** will be discussed in a later section. 

Mirabalin (**86**), initially reported as mirabilin, was isolated from *Siliquariaspongia mirabilis* collected southeast of Chuuk lagoon in the Federated States of Micronesia (Figure 24) [80,81]. **86** is characterized by the presence of a 35-membered macrolide lactam ring bearing a pentadiene conjugated system, a tetra-substituted tetrahydropyran ring, and a linear polyketide moiety attached to the macrolide ring via an amide linkage. **86** inhibited the growth of the HCT-116 cell line with an IC_50_ value of 0.27 µM and was not cytotoxic to several other cell lines tested. **86** is the first macrolide of the chondropsin family with a conjugated pentadiene and a tetrasubstitued tetrahydropyran ring as well as being the first such macrolide to be isolated from a member of the Theonellidae family. Mirabalin was difficult to work with as it was unstable in ambient light and degraded within 3–4 h.

**Figure 25:**
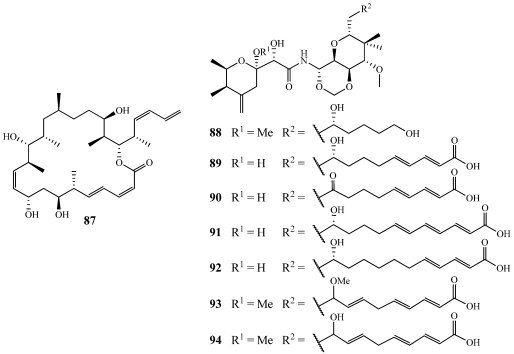
Dictyostatin-1 (**87**) was isolated from a deep-water sponge of the Family Neopeltidae. Theopederin F–J (**88**–**92**) were isolated from a Japanese collection of *Theonella swinhoei*. Theopederin K and L (**93**,**94**) were isolated from deep-water specimens of *Discodermia* sp.

Dictyostatin-1 (**87**) was initially isolated in 1994 from a marine sponge in the genus *Spongia* sp. but in 2003, it was isolated from a Corallistidae (now revised to be Neopeltidae) collected using the Johnson-Sea-Link submersible at a depth of 442 m off the north coast of Jamaica (Figure 25) [82,83]. In initial studies, dictyostatin-1 arrested cells in the G2/M phase of the cell cycle [82]. **87** has been shown to be a potent promoter of tubulin assembly similar to paclitaxel and discodermolide (**133**) [84]. It has recently been synthesized and is also being used in work by the Paterson and Curran/Day Groups to make discodermolide-dictyostatin analogs [85,86,87,88,89,90,91]. 

Theopederin F–J (**88**–**92**) were isolated from *T. swinhoei* collected off the Kerama Islands, Ryukyu Archipelago, Japan (Figure 25) [92]. **88** inhibited the growth of wild type *S. cerevisiae* with an 11 mm inhibitory zone at 10 pg/disk and against the *erg6* mutant with a 12 mm inhibitory zone at 1 µg/disk. **88** was also found to be cytotoxic to the P388 cell line with an IC_50_ value of 0.28 nM. **89**–**92** exhibited similar activities but could not be evaluated due to sample constraints. Theopederins K (**93**) and L (**94**) were isolated from four specimens of *Discodermia* sp. collected by the Johnson-Sea-Link submersible at depths of 121–125 m off the north coast of Honduras (Figure 25) [93]. **93** and **94** exhibited potent *in vitro* cytotoxicity against the P-388 cell line with IC_50_ values of 0.1 and 7.3 nM and the A-549 cell line with IC_50_ values of 1.5 and 3.2 nM, respectively. They are all derivatives of theopederin A and B and mycalamide A with the major difference being alterations in the side chain [94,95]. 

**Figure 26:**
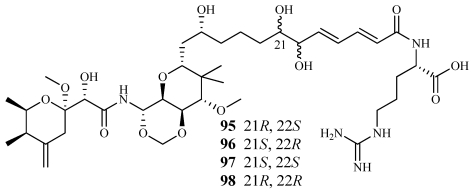
Four onnamide A analogs, 21,22-dihydroxyonnamides A_1_–A_4_ (**95**–**98**), were isolated from an Okinawan collection of *Theonella swinhoei*.

Four new analogs of onnamide A named 21,22-dihydroxyonnamides A_1_–A_4_ (**95**–**98**) were isolated from a polar fraction of *Theonella swinhoei* collected off Okinawa, Japan (Figure 26) [96]. Due to isomerism around the diol, they could only be separated after conversion to isopropylidene derivatives. A 2:2:2:1 isomeric mixture inhibited the P388 cell line with an IC_50_ value of 46 nM, but each one alone did not have the same cytotoxicity. Recently, the Piel group at the University of Bonn, Germany isolated the biosynthetic gene cluster for onnamide A from the metagenome of *Theonella swinhoei* [9]. Their research showed that onnamide A was clearly produced by a bacterial symbiont and not the host sponge [9]. 

## 5. Sterols, Lipids, and Fatty Acids

**Figure 27:**
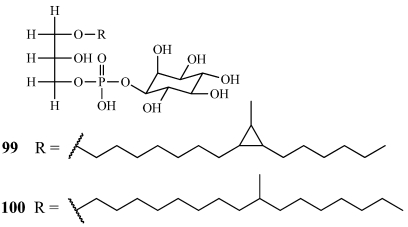
Lysoplasmanylinositols 1 and 2 (**99**,**100**) were isolated from a Japanese collection of *Theonella swinhoei*.

Lysoplasmanylinositols 1 (**99**) and 2 (**100**) were isolated from a collection of *T. swinhoei* with a white interior off Hachijo-jima Island, Japan (Figure 27) [97]. **100** inhibited the growth of *E. coli* at 50 µg/disk with a 12 mm inhibitory zone but activity was only observed for **99** through bioautography. Related lysophosphatidylinositols have been previously isolated from the ascidian *Halocynthia roretzi* as antifungal constituents but this is the first report of lysoplasmanylinositols isolated from a marine organism [98]. 

**Figure 28:**
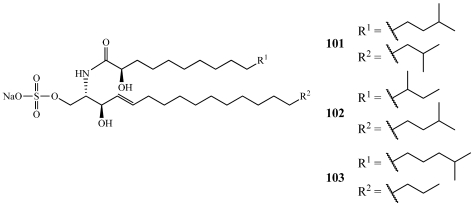
Calyceramides A–C (**101**–**103**) were isolated from a Japanese collection of *Discodermia calyx*.

Calyceramides A–C (**101**–**103**) were isolated from *Discodermia calyx* collected off Sikine-jima Island, Japan (Figure 28) [99]. They are sulfated ceramides that inhibit neuraminidase with IC_50_ values of 0.63, 0.32, and 1.3 µM, respectively. They are very closely related to the ceramide 1-sulfates, which are inhibitors of DNA topoisomerase I isolated from the bryozoan *Watersipora cucullata* [100]. 

**Figure 29:**
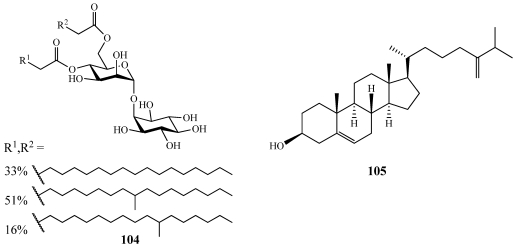
Discoside (**104**) was isolated from a Bahamian collection of *Discodermia dissoluta*. Azoricasterol (**105**) was isolated from a deep-water specimen of *Macandrewia azorica*.

Discoside (**104**) was isolated from *Discodermia dissoluta* collected off the coast of Little San Salvador, Bahamas (Figure 29) [101]. **104** is the first example of a 4,6-*O*-diacylated mannose attached to the 2-hydroxyl group of a *myo*-inositol unit. Compounds similar to **104** have not been reported from marine sponges and the only analogue of **104** reported previously was isolated from various strains of *Propionibacterium* [102].

Azoricasterol (**105**) is the first metabolite isolated from *Macandrewia azorica* (Figure 29) [103]. The specimen was collected from a depth of 600 m off the flanks of the Gettysburg and Ormonde Sea Mount in the North Atlantic by benthic dredging. **105** has an unusual side chain that has two additional methyl groups and a quaternary center forming a rare elongated side chain for sterols. 

**Figure 30:**
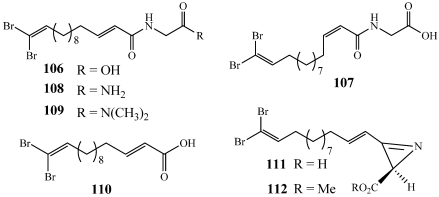
Motualevic acids A–F (**106**–**111**) and (4*E*)-*R*-antazirine (**112**) were isolated from a Fijian collection of *Siliquariaspongia* sp.

Motualevic acids A–F (**106**–**111**) were isolated along with a new enantiomer of antazirine, (4*E*)-*R*-antazirine (**112**), from the marine sponge *Siliquariaspongia* sp. collected on the Motualevu reef in Fiji (Figure 30) [104]. **106**–**109** are the first glycyl conjugates of the *ω*-brominated lipid (*E*)-14,14-dibromotetradeca-2,13-dienoic acid and **111** is the first long-chain 2*H*-azirine 2-carboxylic acid to be found in nature. The carboxylic acid-containing compounds **106** and **111** were found to inhibit the growth of *S. aureus* with MIC_50_ values of 10.9 and 1.2 µg/mL as well as methicillin-resistant *S. aureus* with MIC_50_ values of 21 and 9.3 µg/mL, respectively. 

**Figure 31:**
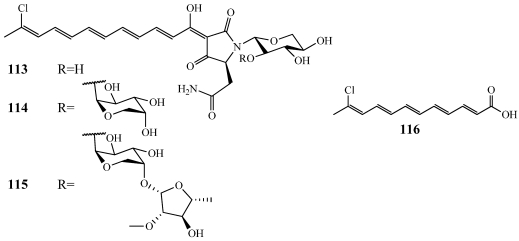
Aurantosides G–I (**113**–**115**) and aurantoic acid (**116**) were isolated from *Theonella swinhoei*.

Aurantosides G–I (**113**–**115**) were isolated from *T. swinhoei* collected in Milne Bay, Papua New Guinea (Figure 31) [105]. They are similar to aurantoside A but have one less chlorinated methylene unit in the polyene side chain [106]. Also, while aurantoside A is a trisaccharide, **113** is a monosaccharide and **114** is a disaccharide. **113**–**115** did not inhibit the HCT-116 colorectal carcinoma cell line when tested at 152, 124, and 103 µM, respectively. They were also inactive in an anti-HIV assay when tested at concentrations of 20, 15, and 12.8 µM, respectively. Aurantoic acid (**116**) is a chlorinated polyene moiety that is evident in the structures of the **113**–**115** (Figure 31) [30]. **116** was isolated from *T. swinhoei* collected at a depth of 20–50 m in Bunaken Marine Park, North Sulawesi, Indonesia along with dehydroconicasterol (**120**, discussed later). **116** showed minimal inhibition when tested at a concentration of 70 µM against C6 glioma, HeLa epithelial carcinoma, and H9c2 cardiac myoblast cell lines. 

**Figure 32:**
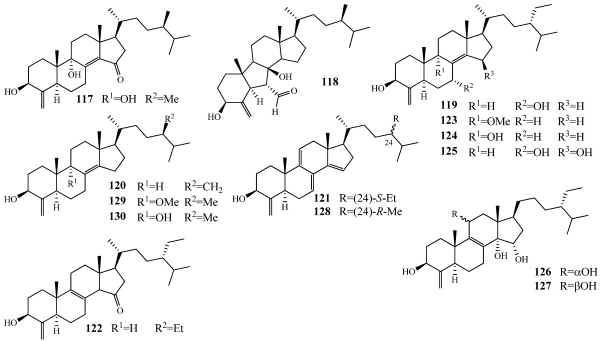
New conicasterol and theonellasterol derivatives (**117**–**130**) isolated from numerous collections of *Theonella swinhoei*.

Sponges of the genus *Theonella* are known to produce biosynthetically unique 4-methylene sterols and these may be ideal taxonomic markers [107]. Recently, a series of conicasterol and theonellasterol derivatives (**117**–**130**) were isolated from *Theonella swinhoei* collected from Yongxing Island in the South China Sea, at Panglao Island, Bohol, Philippines, on the barrier reef of Vangunu and Malaita Island, Solomon Islands, and at Bunaken Marine Park, North Sulawesi, Indonesia (Figure 32) [30,107,108,109]. 9α-hydroxy-15-oxoconicasterol (**117**) and 8β-hydroxy-B-norconicasta-6α-aldehyde (**118**) have novel hydroxyl substitution at either the C-9 or C-8 position and **118** has a B-nor-framework [107]. 7α-hydroxytheonellasterol (**119**) has an additional hydroxyl group at C-7 and an ethyl group at C-24 when compared to the known compounds theonellasterol and swinhosterol C [110,111]. Dehydroconicasterol (**120**) was isolated alongside aurantoic acid (**116**) and showed minimal inhibition when tested at a concentration of 70 µM in C6, HeLa, and H9c2 cell lines [30]. The biological activity of **117** and **118** has not evaluated but **119** showed cytotoxicity against an undisclosed panel of eight cell lines with a mean IC_50_ value of 29.5 µM [109]. Theonellasterols B–H (**121**–**127**) and conicasterols B–D (**128**–**130**) were evaluated for their ability to affect the nuclear receptors, pregnane-X-receptor (PXR) and farnesoid-X-receptor (FXR) [108]. Using a HepG2 human hepatoma reporter cell line transfected with FXR or PXR, **126** partially activated FXR at 10 µM and **121**, **122**, **124**, and **126**–**130** were effective antagonists of FXR transactivation by chenodeoxycholic acid. All of the compounds (**121**–**130**) were agonists of PXR at 10 µM. **126** is one of the first natural products that modulates FXR and is also a ligand for PXR. A molecular docking study was conducted for **121**–**128** and **130** to determine structure-activity relationships [108]. 

**Figure 33:**
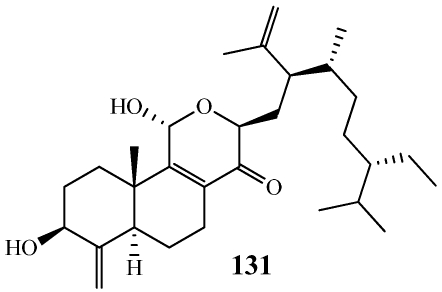
Malaitasterol A (**131**) was isolated from *Theonella swinhoei* collected in the Solomon Islands.

Malaitasterol A (**131**) is an unprecedented secosterol isolated from *Theonella swinhoei* collected at a depth of 22 m off the western coast of Malaita Island, Solomon Islands (Figure 33) [112]. Its unique 11, 12, 13, 14-bis-secosteroid structure was determined by extensive spectroscopic analysis and DFT ^13^C calculations. It is structurally similar to the other known 4-methylene sterols **117**–**130** but its bis-secosteroid structure has not been observed from natural sources. It was found to be a potent inducer of PXR transactivation at a concentration of 10 µM with no effect on FXR. Its PXR activity was confirmed by an increase in the gene expression of downstream PXR targets as well as in molecular docking studies. 

## 6. Alkaloids

**Figure 34:**
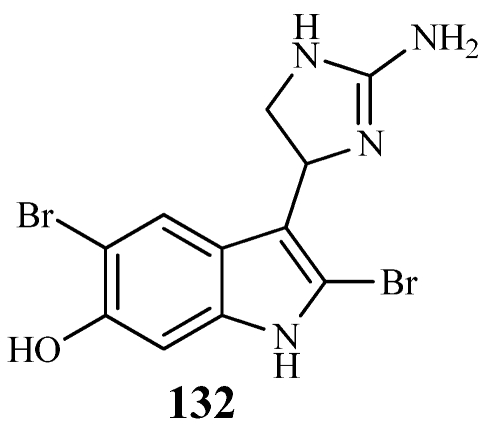
The discodermindole analog, 6-hydroxydiscodermindole (**132**), was isolated from Bahamian collections of *Discodermia polydiscus*.

Collections of *Discodermia polydiscus* from the north point of the Acklins Island, Bahamas and off Grand Bahama Island yielded the discodermindole analog; 6-hydroxydiscodermindole (**132**) in trace amounts (Figure 34) [113]. **132** inhibited the proliferation of the P388 cell line with an IC_50_ value of 12.4 µM. 

## 7. Recent Total Synthesis of Lithistid Natural Products

The total synthesis of a number of lithistid derived compounds has been achieved over the past decade. Some of these syntheses resulted in the reassignment of the initially proposed relative configurations while some allowed for the assignment of absolute configurations. 

**Figure 35:**
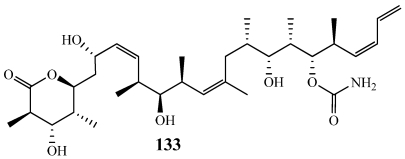
A multi-gram total synthesis of discodermolide (**133**) was completed in order for Novartis to begin Phase I clinical trials.

Discodermolide(**133**) inhibits cell proliferation by polymerizing and hyperstabilizing tubulin similar to paclitaxel but shows activity against paclitaxel resistant tumors (Figure 35). A number of syntheses have been previously reviewed for **133** but the large-scale synthesis accomplished during this decade was very important to provide enough of the compound for clinical trials [5,73,114,115,116]. Despite the other microtubule-stabilizing agents discovered, **133** is the most potent natural promoter of tubulin assembly discovered to date [116,117]. Though clinical trials have been halted, more recent research has shown that discodermolide and paclitaxel have a synergistic effect when used in combination [118]. Some excellent reviews have been published that discuss the different approaches used in the synthesis of discodermolide [85,116,119]. 

There have been a number of total syntheses published on dictyostatin-1 (**87**) [86,91,120,121]. An excellent review was recently published that discusses the different synthetic approaches used in the total synthesis of dictyostatin-1 (**87**) (Figure 25) [85]. In the review, some of the exciting work on the synthesis of discodermolide analogs, dictyostatin-1 analogs, and discodermolide-dictyostatin-1 hybrids is also discussed [84,85,86,87,88,89,90,91,120,122,123,124,125,126,127]. 

**Figure 36:**
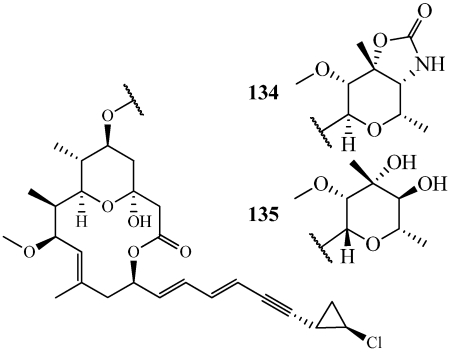
The total syntheses of callipeltoside A (**134**)and C (**135**) were completed with the longest linear sequences of 23 and 20 steps, respectively.

Callipeltoside A (**134**) was isolated from a specimen of *Callipelta* sp. collected off New Caledonia in 1996 (Figure 36) [128]. **134** inhibited the proliferation of the KB and P388 cell lines and, in the NSCLC-N6 non-small-cell bronchopulmonary carcinoma cell line, **134** arrested cells in the G1 phase. Based on its stereochemical and structural complexity coupled with its unique biological activity, members of the callipeltoside family became attractive targets for total synthesis. The first total synthesis of **134** was a convergent synthesis that focused on making three main pieces: the macrolide, the side chain, and the sugar with an overall yield of 0.47% in 50 steps [129]. Other syntheses have been published by the Evans group at Harvard University, the Panek group at Boston University, and the Paterson group [130,131,132]. Their different synthetic approaches increased the overall yield to a maximum of 4.8% with a longest linear sequence of 23 steps [132]. Callipeltoside C (**135**) was recently synthesized resulting in the revision of the absolute configuration around its sugar moiety (Figure 36) [133,134]. It was completed with a longest linear sequence of 20 steps in 11% overall yield. 

**Figure 37:**
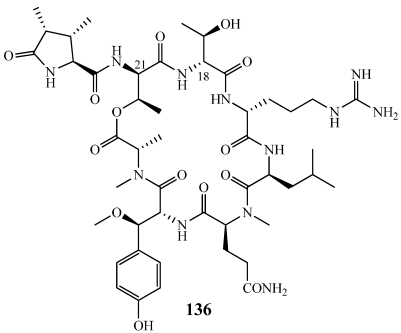
The total synthesis of callipeltin B (**136**) revised its structure from 18*S*, 21*S* to 18*R*, 21*R*.

Callipeltin B (**136**) is an eight-residue cyclic depsipeptide isolated from *Callipelta* sp. collected off New Caledonia (Figure 37) [135]. **136** was found to inhibit the proliferation of the KB, P388, and NSCLC-N6 cell lines. It is structurally similar to callipeltin A whose structure was revised after its original publication although its total synthesis has not been completed [39,136,137]. The total synthesis of **136** was completed using a solid-phase support with a 15% overall yield. By comparing data with the original proposed structure, the configuration of **136** was reassigned from 18*S*, 21*S* to 18*R*, 21*R*. A series of callipeltin analogs have since been synthesized and analyzed for their ability to inhibit the HeLa cervical adenocarcinoma cell line [138]. 

**Figure 38:**
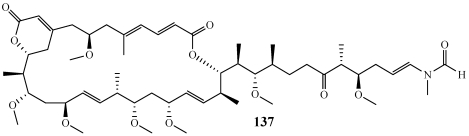
The absolute configuration of reidispongiolide A (**137**) was determined through total synthesis.

Reidispongiolide A (**137**) is a polyketide containing a 26-member macrolide ring which was isolated from a specimen of *Reidispongia coerulea* collected off New Caledonia (Figure 38) [139,140]. It is a member of the sphinxolide/reidispongiolide class of macrolides which are members of an emerging class of actin-binding cytotoxic macrolides [139]. The absolute configuration of reidispongiolide A had not been determined; therefore the Paterson group carried out the total synthesis defining it as shown in **137**. They completed the total synthesis with a longest linear sequence of 25 steps in a 1.1% overall yield [141]. 

Neopeltolide (**85**) has received significant attention from synthetic chemists since its publication in 2007 due to its interesting structural aspects and potent biological activity (Figure 24) [76]. The first total synthesis of **85** was completed by the Panek group and had a longest linear sequence of 19 steps with an overall yield of 1.3% [78]. It was determined from this initial synthesis that the stereochemistry, which was originally proposed as 11*R*, 13*R*, is actually 11*S*, 13*S*. A number of syntheses followed and all were in agreement with the Panek group’s initial observation [77,78,79,142,143,144]. The highest yielding synthesis to date was reported by the Fuwa/Sasaki group at Tohuku University which prepared **85** with the longest linear sequence of 25 steps and an 8.3% overall yield [142]. Synthetic neopeltolide analogs have been tested against a variety of human, murine, and bovine cell lines to obtain valuable SAR information [145]. 

**Figure 39:**
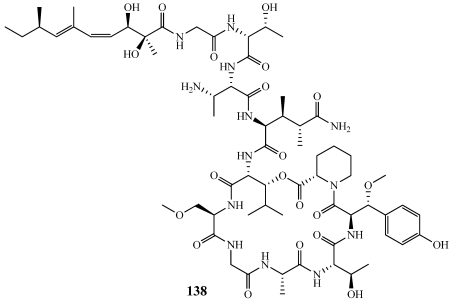
The absolute configuration of papuamide B (**138**) was revised based on the total synthesis.

Papuamide B (**138**) is a 22-membered cyclic depsipeptide connected to a complex linear tetrapeptide via an amide linkage (Figure 39) [16]. Both papuamide A (**16**) and B (**138**) exhibit a potent inhibitory effect on the infection of human T-lymphoblastoid cells by HIV-1_RF_ with an EC_50_ value of 4 ng/mL. The total synthesis of **138** was completed by the Ma Group at the Chinese Academy of Sciences and suggested a revision of the 2,3-diaminobutanoic acid in the side chain from 2*S*, 3*R* to 2*S*, 3*S* [146]. The papuamides are structural analogs of the mirabamides (**8**–**15**)and have very similar biological activities. This synthetic scheme is amenable to complete the total synthesis of closely related compounds as well as determine SAR information. 

**Figure 40:**
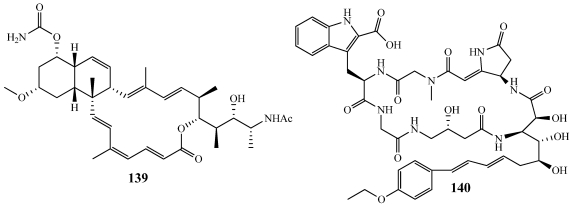
The total synthesis of superstolide A (**139**) and microsclerodermin E (**140**) have recently been achieved.

Superstolide A (**139**) is a macrolide isolated from *Neosiphonia superstes* collected off New Caledonia (Figure 40) [147]. It is highly cytotoxic against several cancer cell lines including the P388, human nasopharyngeal KB, and NSCLC-N6-L16 non-small cell lung carcinoma cell lines with IC_50_ values of 5, 8, and 6 nM, respectively [147]. Due to their interesting chemical structures and potent biological properties, the superstolides have attracted considerable attention as synthetic targets. The Roush group at Scripps Florida reported the total synthesis in 2008 [148]. Their synthetic route was likely biomimetic in nature and utilized an intramolecular Suzuki coupling reaction and a highly stereoselective transannular Diels-Alder cycloaddition [148]. 

Microsclerodermin E (**140**) and its series of cyclic peptides were isolated by Faulkner and co-workers at Scripps Institute of Oceanography (Figure 40) [24]. Structurally, each of them contains several unique features, including a 23-membered cyclic hexapeptide core, featuring a very complex *β*-amino acid and three other unnatural amino acid residues. The Ma group at the Chinese Academy of Sciences reported the first total synthesis of a member of the microsclerodermin group with a 1% overall yield in 26 linear steps [149].

Scleritodermin A (**29**) was recently isolated from *Scleritoderma* *nodosum* (Figure 7) [27]. It has an interesting structure and was found to have potent biological activities as well as cause cell cycle arrest in the G2/M phase. The total synthesis was undertaken by the Nan group at the Chinese Academy of Sciences and the structure was revised from 2*Z*, 4*E* and 14*R* to 2*E*, 4*E* and 14*S* [28]. 

**Figure 41:**
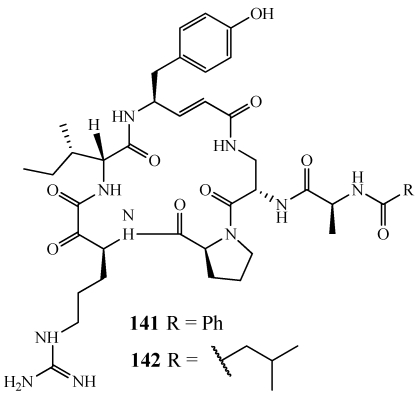
Cyclotheonamides E2 and E3 (**141**,**142**) are potent inhibitors of serine proteases and their total synthesis has been completed.

Cyclotheonamides E2 (**141**) and E3 (**142**) are cyclic pentapeptides found to be potent inhibitors of serine proteases (Figure 41) [150]. Because of their biological properties and unusual structural features, further research was done on their mode of enzyme inhibition as well as their total synthesis. **141** and **142** are structurally similar to cyclotheonamides A and B, both of which have been synthesized previously [151,152,153]. The differences are a D-alloisoleucine in place of D-phenylalanine as well as the addition of the benzoylalanine and isovalerylalanine side chain [151]. The Wasserman group at Yale University synthesized **141** and **142** using a cyano ylide method [154]. 

Miraziridine A (**57**) is structurally similar to known compounds that are inhibitors of trypsin-like serine proteases, papain-like cysteine proteases, and pepsin-like aspartyl proteases (Figure 13) [49]. The total synthesis of **57** was completed by Schaschke at the Max-Planck-Institut für Biochemie in order to use **57** as a blueprint to efficiently design small molecule protease inhibitors [51]. The Konno group at the Kyoto Prefectural University of Medicine produced **57** as well as a number of analogs to provide important SAR information [50]. They determined that the N-terminal aziridine is important for cathepsin B inhibition.

Leiodolide B (**78**) was recently isolated from a deep-water sponge *Leiodermatium* sp. by the Fenical group (Figure 21) [69]. Since only a very small amount (0.8 mg) could be isolated from the natural source, its structure was determined based on comparisions with leiodolide A (**77**) and a proposed biosynthetic closure of the brominated tetrahydrofuran ring [69]. The Fürstner group at the Max-Planck-Institut für Kohlenforschung prepared four isomers of **78** yet none of them matched the NMR data of the natural product [70]. Further work is being performed by the Fürstner group to synthesize leiodolides A and B. 

Bitungolide F (**76**) was isolated from an Indonesian collection of *Theonella* cf. *swinhoei* (Figure 20) [65]. **76** is structurally similar to pironetin, a compound isolated from *Streptomyces* sp. which arrests cell at the M-phase of the cell cycle [65]. The structure of bitungolide A (**71**) had been confirmed by X-ray and the structure of **76** was based on comparison with **71**. The She group from Lanzhou University, China completed the total synthesis of **76** in 17 steps with a yield of 20.1% [68]. 

## 8. Conclusion

From the unique activities and amazing diversity of the compounds isolated just over the past decade, sponges from the Order Lithistida will almost certainly continue to be a significant source of interesting biologically active compounds. Recent research has shown that some of the compounds isolated from sponges within this order are likely produced by the microorganisms residing within them and work is ongoing to culture these microbes [1,7,67]. A future area of research will be to understand what unique characteristics of lithistid sponges leads to such a great diversity of microbes and ultimately such a diverse array of compounds.

## References

[1] Bewley C.A., Faulkner D.J. (1998). Lithistid sponges: Star performers or hosts to the stars. Angew. Chem. Int. Ed..

[2] Hooper J.N.A., van Soest R.W.M. (2002). Systema Porifera: A Guide to the Classification of Sponges.

[3] van Soest R.W.M., Boury-Esnault N., Hooper J.N.A., Rützler K., de Voogd N.J., Alvarez B., Hajdu E., Pisera A.B., Vacelet J., Manconi R. World Porifera Database. http://www.marinespecies.org/porifera.

[4] Catalogue of Life: 2007 Annual Checklist: Species 2000 & ITIS Catalogue of Life Hierarchy. http://www.gbif.net.

[5] D’Auria M.V., Zampella A., Zollo F., Atta-ur-Rahman (2002). The Chemistry of Lithistid Sponge: A Spectacular Source of New Metabolites. Bioactive Natural Products.

[6] Fusetani N., Matsunaga S. (1993). Bioactive sponge peptides. Chem. Rev..

[7] Andrianasolo E.H., Gross H., Goeger D., Musafija-Girt M., McPhail K., Leal R.M., Mooberry S.L., Gerwick W.H. (2005). Isolation of swinholide A and related glycosylated derivatives from two field collections of marine cyanobacteria. Org. Lett..

[8] Piel J. (2009). Metabolites from symbiotic bacteria. Nat. Prod. Rep..

[9] Piel J., Hui D., Wen G., Butzke D., Platzer M., Fusetani N., Matsunaga S. (2004). Antitumor polyketide biosynthesis by an uncultivated bacterial symbiont of the marine sponge *Theonella swinhoei*. Proc. Natl. Acad. Sci. USA.

[10] Wright A.E. (2010). The Lithistida: Important sources of compounds useful in biomedical research. Curr. Opin. Biotechnol..

[11] Buckingham J. (2011). Dictionary of Natural Products on DVD.

[12] Ratnayake A.S., Bugni T.S., Feng X., Harper M.K., Skalicky J.J., Mohammed K.A., Andjelic C.D., Barrows L.R., Ireland C.M. (2006). Theopapuamide, a cyclic depsipeptide from a Papua New Guinea Lithistid sponge *Theonella swinhoei*. J. Nat. Prod..

[13] Plaza A., Bifulco G., Keffer J.L., Lloyd J.R., Baker H.L., Bewley C.A. (2008). Celebesides A–C and theopapuamides B–D, depsipeptides from an Indonesian sponge that inhibit HIV-1 entry. J. Org. Chem..

[14] Plaza A., Gustchina E., Baker H.L., Kelly M., Bewley C.A. (2007). Mirabamides A–D, depsipeptides from the sponge *Siliquariaspongia mirabilis* that inhibit HIV-1 fusion. J. Nat. Prod..

[15] Lu Z., van Wagoner R.M., Harper M.K., Baker H.L., Hooper J.N.A., Bewley C.A., Ireland C.M. (2011). Mirabamides E–H, HIV-inhibitory depsipeptides from the sponge *Stelletta clavosa*. J. Nat. Prod..

[16] Ford P.W., Gustafson K.R., McKee T.C., Shigematsu N., Maurizi L.K., Pannell L.K., Williams D.E., Dilip de Silva E., Lassota P., Allen T.M. (1999). Papuamides A–D, HIV-inhibitory and cytotoxic depsipeptides from the sponges *Theonella mirabilis* and *Theonella swinhoei* collected in Papua New Guinea. J. Am. Chem. Soc..

[17] Andjelic C., Planelles V., Barrows L. (2008). Characterizing the anti-HIV activity of papuamide A. Mar. Drugs.

[18] Roy M.C., Ohtani I.I., Ichiba T., Tanaka J., Satari R., Higa T. (2000). New cyclic peptides from the Indonesian sponge *Theonella swinhoei*. Tetrahedron.

[19] Roy M.C., Ohtani I.I., Tanaka J., Higa T., Satari R. (1999). Barangamide A, a new cyclic peptide from the Indonesian sponge *Theonella swinhoei*. Tetrahedron Lett..

[20] Kobayashi M., Lee N.K., Shibuya H., Momose T., Kitagawa I. (1991). Marine natural products. XXVI. Biologically active tridecapeptide lactones from the Okinawan marine sponge *Theonella swinhoei* (Theonellidae). Structures of theonellapeptolides Ia, Ib, Ic, and Ie. Chem. Pharm. Bull..

[21] Green C.J. (1981). Immunosuppression with cyclosporin A: A review. Diagn. Histopathol..

[22] Okada Y., Matsunaga S., van Soest R.W.M., Fusetani N. (2002). Nagahamide A, an antibacterial depsipeptide from the marine sponge *Theonella swinhoei*. Org. Lett..

[23] Bewley C.A., Debitus C., Faulkner D.J. (1994). Microsclerodermins A and B. Antifungal cyclic peptides from the Lithistid sponge *Microscleroderma* sp.. J. Am. Chem. Soc..

[24] Schmidt E.W., Faulkner D.J. (1998). Microsclerodermins C–E, antifungal cyclic peptides from the lithistid marine sponges *Theonella* sp. and *Microscleroderma* sp.. Tetrahedron.

[25] Qureshi A., Colin P.L., Faulkner D.J. (2000). Microsclerodermins F–I, antitumor and antifungal cyclic peptides from the Lithistid sponge *Microscleroderma* sp.. Tetrahedron.

[26] Erdogan I., Tanaka J.I., Higa T. (2000). Two cyclic hexapeptides from the marine sponge *Theonella cupola*. FABAD J. Pharm. Sci..

[27] Schmidt E.W., Raventos-Suarez C., Bifano M., Menendez A.T., Fairchild C.R., Faulkner D.J. (2004). Scleritodermin A, a cytotoxic cyclic peptide from the Lithistid sponge *Scleritoderma nodosum*. J. Nat. Prod..

[28] Liu S., Cui Y., Nan F. (2008). Total synthesis of the originally proposed and revised structures of scleritodermin A. Org. Lett..

[29] Sellanes D., Manta E., Serra G. (2007). Toward the total synthesis of scleritodermin A: Preparation of the C1-N15 fragment. Tetrahedron Lett..

[30] Angawi R.F., Calcinai B., Cerrano C., Dien H.A., Fattorusso E., Scala F., Taglialatela-Scafati O. (2009). Dehydroconicasterol and aurantoic acid, a chlorinated polyene derivative, from the Indonesian sponge *Theonella swinhoei*. J. Nat. Prod..

[31] Carmely S., Kashman Y. (1985). Structure of swinholide A, a new macrolide from the marine sponge. Tetrahedron Lett..

[32] De Silva E.D., Williams D.E., Andersen R.J., Klix H., Holmes C.F.B., Allen T.M. (1992). Motuporin, a potent protein phosphatase inhibitor isolated from the Papua New Guinea sponge *Theonella swinhoei*. Tetrahedron Lett..

[33] Klenchin V.A., King R., Tanaka J., Marriott G., Rayment I. (2005). Structural basis of swinholide A binding to actin. Chem. Biol..

[34] Maynes J.T., Luu H.A., Cherney M.M., Andersen R.J., Williams D., Holmes C.F.B., James M.N.G. (2006). Crystal structures of protein phosphatase-1 bound to motuporin and dihydromicrocystin-LA: Elucidation of the mechanism of enzyme inhibition by cyanobacterial toxins. J. Mol. Biol..

[35] Wegerski C.J., Hammond J., Tenney K., Matainaho T., Crews P. (2006). A serendipitous discovery of isomotuporin-containing sponge populations of *Theonella swinhoei*. J. Nat. Prod..

[36] Zampella A., Sepe V., Bellotta F., Luciano P., D’Auria M.V., Cresteil T., Debitus C., Petek S., Poupat C., Ahond A. (2009). Homophymines B–E and A1–E1, a family of bioactive cyclodepsipeptides from the sponge *Homophymia* sp.. Org. Biomol. Chem..

[37] Zampella A., Sepe V., Luciano P., Bellotta F., Monti M.C., D’Auria M.V., Jepsen T., Petek S., Adeline M.-T., Laprevote O. (2008). Homophymine A, an anti-HIV cyclodepsipeptide from the sponge *Homophymia* sp.. J. Org. Chem..

[38] Oku N., Gustafson K.R., Cartner L.K., Wilson J.A., Shigematsu N., Hess S., Pannell L.K., Boyd M.R., McMahon J.B. (2004). Neamphamide A, a new HIV-inhibitory depsipeptide from the Papua New Guinea marine sponge *Neamphius huxleyi*. J. Nat. Prod..

[39] Zampella A., D’Auria M.V., Paloma L.G., Casapullo A., Minale L., Debitus C., Henin Y. (1996). Callipeltin A, an anti-HIV cyclic depsipeptide from the New Caledonian Lithistida sponge *Callipelta* sp.. J. Am. Chem. Soc..

[40] Plaza A., Keffer J.L., Lloyd J.R., Colin P.L., Bewley C.A. (2010). Paltolides A–C, anabaenopeptin-type peptides from the Palau sponge *Theonella swinhoei*. J. Nat. Prod..

[41] Bjoerquist P., Buchanan M., Campitelli M., Carroll A., Hyde E., Neve J., Polla M., Quinn R. (2008). Use of cyclic anabaenopeptin-type peptides for the treatment of a condition wherein inhibition of carboxypeptidase U is beneficial, novel anabaenopeptin derivatives and intermediates thereof. PCT Int. Appl..

[42] Plaza A., Bifulco G., Masullo M., Lloyd J.R., Keffer J.L., Colin P.L., Hooper J.N.A., Bell L.J., Bewley C.A. (2010). Mutremdamide A and koshikamides C–H, peptide inhibitors of HIV-1 entry from different *Theonella* species. J. Org. Chem..

[43] Gulavita N.K., Pomponi S.A., Wright A.E., Yarwood D., Sills M.A. (1994). Isolation and structure elucidation of perthamide B, a novel peptide from the sponge *Theonella* sp.. Tetrahedron Lett..

[44] Festa C., de Marino S., Sepe V., Monti M.C., Luciano P., D’Auria M.V., Débitus C., Bucci M., Vellecco V., Zampella A. (2009). Perthamides C and D, two new potent anti-inflammatory cyclopeptides from a Solomon Lithistid sponge *Theonella swinhoei*. Tetrahedron.

[45] Sepe V., D’Auria M.V., Bifulco G., Ummarino R., Zampella A. (2010). Concise synthesis of AHMHA unit in perthamide C. Structural and stereochemical revision of perthamide C. Tetrahedron.

[46] Festa C., de Marino S., Sepe V., D’Auria M.V., Bifulco G., Andrés R., Terencio M.C., Payá M., Debitus C., Zampella A. (2011). Perthamides C–F, potent human antipsoriatic cyclopeptides. Tetrahedron.

[47] Araki T., Matsunaga S., Nakao Y., Furihata K., West L., Faulkner D.J., Fusetani N. (2008). Koshikamide B, a cytotoxic peptide lactone from a marine sponge *Theonella* sp.. J. Org. Chem..

[48] Festa C., de Marino S., Sepe V., D’Auria M.V., Bifulco G., Debitus C., Bucci M., Vellecco V., Zampella A. (2011). Solomonamides A and B, new anti-inflammatory peptides from *Theonella swinhoei*. Org. Lett..

[49] Nakao Y., Fujita M., Warabi K., Matsunaga S., Fusetani N. (2000). Miraziridine A, a novel cysteine protease inhibitor from the marine sponge *Theonella* aff. *mirabilis*. J. Am. Chem. Soc..

[50] Konno H., Kubo K., Makabe H., Toshiro E., Hinoda N., Nosaka K., Akaji K. (2007). Total synthesis of miraziridine A and identification of its major reaction site for cathepsin B. Tetrahedron.

[51] Schaschke N. (2004). Miraziridine A: Natures blueprint towards protease class-spanning inhibitors. Bioorg. Med. Chem. Lett..

[52] Araki T., Matsunaga S., Fusetani N. (2005). Koshikamide A2, a cytotoxic linear undecapeptide isolated from a marine sponge of *Theonella* sp.. Biosci. Biotechnol. Biochem..

[53] Hamada T., Matsunaga S., Yano G., Fusetani N. (2004). Polytheonamides A and B, highly cytotoxic, linear polypeptides with unprecedented structural features, from the marine sponge, *Theonella swinhoei*. J. Am. Chem. Soc..

[54] Hamada T., Sugawara T., Matsunaga S., Fusetani N. (1994). Polytheonamides, unprecedented highly cytotoxic polypeptides from the marine sponge *Theonella swinhoei*. Structure elucidation. Tetrahedron Lett..

[55] Ketchem R.R., Lee K.C., Huo S., Cross T.A. (1996). Macromolecular structural elucidation with solid-state NMR-derived orientational constraints. J. Biomol. NMR.

[56] Youssef D.T.A., Mooberry S.L. (2006). Hurghadolide A and swinholide I, potent actin-microfilament disrupters from the Red Sea sponge *Theonella swinhoei*. J. Nat. Prod..

[57] Kobayashi M., Tanaka J., Katori T., Yamashita M., Kitagawa I. (1990). Marine natural products. XXII.: The absolute stereostructure of swinholide A, a potent cytotoxic dimeric macrolide from the Okinawan marine sponge *Theonella swinhoei*. Chem. Pharm. Bull..

[58] Marino S.D., Festa C., D’Auria M.V., Cresteil T., Debitus C., Zampella A. (2011). Swinholide J, a potent cytotoxin from the marine sponge *Theonella swinhoei*. Mar. Drugs.

[59] Edrada R.A., Ebel R., Supriyono A., Wray V., Schupp P., Steube K., van Soest R., Proksch P. (2002). Swinhoeiamide A, a new highly active calyculin derivative from the marine sponge *Theonella swinhoei*. J. Nat. Prod..

[60] Matsunaga S., Wakimoto T., Fusetani N. (1997). Isolation of four new calyculins from the marine sponge *Discodermia calyx*. J. Org. Chem..

[61] Wakimoto T., Matsunaga S., Takai A., Fusetani N. (2002). Insight into binding of calyculin A to protein phosphatase 1: Isolation of hemicalyculin A and chemical transformation of calyculin A. Chem. Biol..

[62] Bagu J.R., Sykes B.D., Craig M.M., Holmes C.F.B. (1997). A molecular basis for different interactions of marine toxins with protein phosphatase-1. J. Biol. Chem..

[63] Kita A., Matsunaga S., Takai A., Kataiwa H., Wakimoto T., Fusetani N., Isobe M., Miki K. (2002). Crystal structure of the complex between calyculin A and the catalytic subunit of protein phosphatase 1. Structure.

[64] Lindvall M.K., Pihko P.M., Koskinen A.M.P. (1997). The binding mode of calyculin A to protein phosphatase-1. J. Biol. Chem..

[65] Sirirath S., Tanaka J., Ohtani I.I., Ichiba T., Rachmat R., Ueda K., Usui T., Osada H., Higa T. (2002). Bitungolides A–F, new polyketides from the Indonesian sponge *Theonella* cf. *swinhoei*. J. Nat. Prod..

[66] Kobayashi S., Tsuchiya K., Kurokawa T., Nakagawa T., Shimada N., Iitaka Y. (1994). Pironetin, a novel plant growth regulator produced by *Streptomyces* sp. NK10958. II. Structure elucidation. J. Antibiot..

[67] Kondoh M., Usui T., Kobayashi S., Tsuchiya K., Nishikawa K., Nishikiori T., Mayumi T., Osada H. (1998). Cell cycle arrest and antitumor activity of pironetin and its derivatives. Cancer Lett..

[68] Su Y., Xu Y., Han J., Zheng J., Qi J., Jiang T., Pan X., She X. (2009). Total Synthesis of (−)-Bitungolide F. J. Org. Chem..

[69] Sandler J.S., Colin P.L., Kelly M., Fenical W. (2006). Cytotoxic macrolides from a new species of the deep-water marine sponge *Leiodermatium*. J. Org. Chem..

[70] Larivee A., Unger J.B., Thomas M., Wirtz C., Dubost C., Handa S., Fürstner A. (2011). The leiodolide B puzzle. Angew. Chem. Int. Ed..

[71] Paterson I., Dalby S.M., Roberts J.C., Naylor G.J., Guzman E.A., Isbrucker R., Pitts T.P., Linley P., Divlianska D., Reed J.K. (2011). Leiodermatolide, a potent antimitotic macrolide from the marine sponge *Leiodermatium* sp.. Angew. Chem. Int. Ed..

[72] Gunasekera S.P., Paul G.K., Longley R.E., Isbrucker R.A., Pomponi S.A. (2002). Five new discodermolide analogues from the marine sponge *Discodermia* sp.. J. Nat. Prod..

[73] Gunasekera S.P., Gunasekera M., Longley R.E., Schulte G.K. (1990). Discodermolide: A new bioactive polyhydroxylated lactone from the marine sponge *Discodermia dissoluta*. J. Org. Chem..

[74] Gunasekera S.P., Longley R.E., Isbrucker R.A. (2001). Acetylated analogues of the microtubule-stabilizing agent discodermolide: Preparation and biological activity. J. Nat. Prod..

[75] Isbrucker R., Gunasekera S., Longley R. (2001). Structure-activity relationship studies of discodermolide and its semisynthetic acetylated analogs on microtubule function and cytotoxicity. Cancer Chemother. Pharmacol..

[76] Wright A.E., Botelho J.C., Guzman E., Harmody D., Linley P., McCarthy P.J., Pitts T.P., Pomponi S.A., Reed J.K. (2007). Neopeltolide, a macrolide from a Lithistid sponge of the Family Neopeltidae. J. Nat. Prod..

[77] Ulanovskaya O.A., Janjic J., Suzuki M., Sabharwal S.S., Schumacker P.T., Kron S.J., Kozmin S.A. (2008). Synthesis enables identification of the cellular target of leucascandrolide A and neopeltolide. Nat Chem. Biol..

[78] Youngsaye W., Lowe J.T., Pohlki F., Ralifo P., Panek J.S. (2007). Total synthesis and stereochemical reassignment of (+)-neopeltolide. Angew. Chem. Int. Ed..

[79] Custar D.W., Zabawa T.P., Scheidt K.A. (2008). Total synthesis and structural revision of the marine macrolide neopeltolide. J. Am. Chem. Soc..

[80] Plaza A., Baker H.L., Bewley C.A. (2008). Mirabilin, an antitumor macrolide lactam from the marine sponge *Siliquariaspongia mirabilis*. J. Nat. Prod..

[81] Plaza A., Baker H.L., Bewley C.A. (2009). Mirabalin, an antitumor macrolide lactam from the marine sponge *Siliquariaspongia mirabilis*. J. Nat. Prod..

[82] Isbrucker R.A., Cummins J., Pomponi S.A., Longley R.E., Wright A.E. (2003). Tubulin polymerizing activity of dictyostatin-1, a polyketide of marine sponge origin. Biochem. Pharmacol..

[83] Pettit G.R., Cichacz Z.A., Gao F., Boyd M.R., Schmidt J.M. (1994). Isolation and structure of the cancer cell growth inhibitor dictyostatin-1. Chem. Commun..

[84] Paterson I., Gardner N.M., Guzmán E., Wright A.E. (2009). Total synthesis and biological evaluation of novel C2-C6 region analogues of dictyostatin. Bioorg. Med. Chem..

[85] Florence G.J., Gardner N.M., Paterson I. (2008). Development of practical syntheses of the marine anticancer agents discodermolide and dictyostatin. Nat. Prod. Rep..

[86] Paterson I., Britton R., Delgado O., Meyer A., Poullennec K.G. (2004). Total synthesis and configurational assignment of (−)-dictyostatin, a microtubule-stabilizing macrolide of marine sponge origin. Angew. Chem. Int. Ed..

[87] Paterson I., Naylor G.J., Fujita T., Guzman E., Wright A.E. (2010). Total synthesis of a library of designed hybrids of the microtubule-stabilising anticancer agents taxol, discodermolide and dictyostatin. Chem. Commun. (Camb.).

[88] Paterson I., Naylor G.J., Gardner N.M., Guzman E., Wright A.E. (2011). Total synthesis and biological evaluation of a series of macrocyclic hybrids and analogues of the antimitotic natural products dictyostatin, discodermolide, and taxol. Chem. Asian J..

[89] Paterson I., Naylor G.J., Wright A.E. (2008). Total synthesis of a potent hybrid of the anticancer natural products dictyostatin and discodermolide. Chem. Commun. (Camb.).

[90] Shin Y., Choy N., Balachandran R., Madiraju C., Day B.W., Curran D.P. (2002). Discodermolide/dictyostatin hybrids: Synthesis and biological evaluation. Org. Lett..

[91] Shin Y., Fournier J.-H., Fukui Y., Brückner A.M., Curran D.P. (2004). Total synthesis of (−)-dictyostatin: Confirmation of relative and absolute configurations. Angew. Chem. Int. Ed..

[92] Tsukamoto S., Matsunaga S., Fusetani N., Toh-e A. (1999). Theopederins F–J: Five new antifungal and cytotoxic metabolites from the marine sponge, *Theonella swinhoei*. Tetrahedron.

[93] Paul G.K., Gunasekera S.P., Longley R.E., Pomponi S.A. (2001). Theopederins K and L. Highly potent cytotoxic metabolites from a marine sponge *Discodermia* sp.. J. Nat. Prod..

[94] Fusetani N., Sugawara T., Matsunaga S. (1992). Bioactive marine metabolites. 41. Theopederins A–E, potent antitumor metabolites from a marine sponge, *Theonella* sp.. J. Org. Chem..

[95] Perry N.B., Blunt J.W., Munro M.H.G., Pannell L.K. (1988). Mycalamide A, an antiviral compound from a New Zealand sponge of the genus *Mycale*. J. Am. Chem. Soc..

[96] Miyata Y., Matsunaga S. (2008). Structure elucidation of 21,22-dihydroxyonnamides A1–A4 from the marine sponge *Theonella swinhoei*: An empirical rule to assign the relative stereochemistry of linear 1,5-diols. Tetrahedron Lett..

[97] Matsunaga S., Nishimura S., Fusetani N. (2001). Two new antimicrobial Lysoplasmanylinositols from the marine sponge *Theonella swinhoei*. J. Nat. Prod..

[98] Tsukamoto S., Hirota H., Kato H., Fusetani N. (1993). Isolation of eicosapentaenoyl and arachidonoyl lysophosphatidylinositols from the ascidian *Halocynthia roretzi*. Comp. Biochem. Physiol. Part C.

[99] Nakao Y., Takada K., Matsunaga S., Fusetani N. (2001). Calyceramides A–C: Neuraminidase inhibitory sulfated ceramides from the marine sponge *Discodermia calyx*. Tetrahedron.

[100] Ojika M., Yoshino G., Sakagami Y. (1997). Novel ceramide 1-sulfates, potent DNA topoisomerase I inhibitors isolated from the bryozoa *Watersipora cucullata*. Tetrahedron Lett..

[101] Barbieri L., Costantino V., Fattorusso E., Mangoni A. (2005). Glycolipids from sponges. Part 16. Discoside, a rare myo-inositol-containing glycolipid from the Caribbean sponge *Discodermia dissoluta*. J. Nat. Prod..

[102] Prottey C., Ballou C.E. (1968). Diacyl myoinositol monomannoside from *Propionibacterium shermanii*. J. Biol. Chem..

[103] Gross H., Reitner J., Koenig G.M. (2004). Isolation and structure elucidation of azoricasterol, a new sterol of the deepwater sponge *Macandrewia azorica*. Naturwissenschaften.

[104] Keffer J.L., Plaza A., Bewley C.A. (2009). Motualevic acids A–F, antimicrobial acids from the sponge *Siliquariaspongia* sp.. Org. Lett..

[105] Ratnayake A.S., Davis R.A., Harper M.K., Veltri C.A., Andjelic C.D., Barrows L.R., Ireland C.M. (2004). Aurantosides G, H, and I: Three new tetramic acid glycosides from a Papua New Guinea *Theonella swinhoei*. J. Nat. Prod..

[106] Matsunaga S., Fusetani N., Kato Y., Hirota H. (1991). Aurantosides A and B: Cytotoxic tetramic acid glycosides from the marine sponge *Theonella* sp.. J. Am. Chem. Soc..

[107] Zhang H.J., Yi Y.H., Lin H.W. (2010). Oxygenated 4-methylidene sterols from the South China Sea sponge *Theonella swinhoei*. Helv. Chim. Acta.

[108] De Marino S., Ummarino R., D’Auria M.V., Chini M.G., Bifulco G., Renga B., D’Amore C., Fiorucci S., Debitus C., Zampella A. (2011). Theonellasterols and conicasterols from *Theonella swinhoei*. Novel marine natural ligands for human nuclear receptors. J. Med. Chem..

[109] Qureshi A., Faulkner D.J. (2000). 7α-Hydroxytheonellasterol, a cytotoxic 4-methylene sterol from the Philippines sponge *Theonella swinhoei*. J. Nat. Prod..

[110] Kho E., Imagawa D.K., Rohmer M., Kashman Y., Djerassi C. (1981). Sterols in marine invertebrates. 22. Isolation and structure elucidation of conicasterol and theonellasterol, two new 4-methylene sterols from the Red Sea sponges *Theonella conica* and *Theonella swinhoei*. J. Org. Chem..

[111] Umeyama A., Shoji N., Enoki M., Arihara S. (1997). Swinhosterols A–C, 4-methylene secosteroids from the marine sponge *Theonella swinhoei*. J. Nat. Prod..

[112] de Marino S., Sepe V., D’Auria M.V., Bifulco G., Renga B., Petek S., Fiorucci S., Zampella A. (2011). Towards new ligands of nuclear receptors. Discovery of malaitasterol A, an unique bis-secosterol from marine sponge *Theonella swinhoei*. Org. Biomol. Chem..

[113] Cohen J., Paul G., Gunasekera S., Longley R., Pomponi S. (2004). 6-Hydroxydiscodermindole, A new discodermindole from the marine sponge *Discodermia polydiscus*. Pharmaceut. Biol..

[114] Mickel S.J. (2007). Total Synthesis of the marine natural product (+)-discodermolide in multigram quantities. Pure Appl. Chem..

[115] Smith A.B., Kaufman M.D., Beauchamp T.J., LaMarche M.J., Arimoto H. (1999). Gram-scale synthesis of (+)-discodermolide. Org. Lett..

[116] Smith A.B., Freeze B.S. (2008). (+)-Discodermolide: Total synthesis, construction of novel analogues, and biological evaluation. Tetrahedron.

[117] Buey R.M., Barasoain I., Jackson E., Meyer A., Giannakakou P., Paterson I., Mooberry S., Andreu J.M., Díaz J.F. (2005). Microtubule interactions with chemically diverse stabilizing agents: Thermodynamics of binding to the paclitaxel site predicts cytotoxicity. Chem. Biol..

[118] Martello L., McDaid H., Regl D., Yang C., Meng D., Pettus T., Kaufman M., Arimoto H., Danishefsky S., Smith A. (2000). Taxol and discodermolide represent a synergistic drug combination in human carcinoma cell lines. Clin. Cancer Res..

[119] Shaw S.J. (2008). The structure activity relationship of discodermolide analogues. Mini Rev. Med. Chem..

[120] O’Neil G.W., Phillips A.J. (2006). Total synthesis of (−)-dictyostatin. J. Am. Chem. Soc..

[121] Ramachandran P.V., Srivastava A., Hazra D. (2006). Total synthesis of potential antitumor agent, (−)-dictyostatin. Org. Lett..

[122] Paterson I., Gardner N.M. (2007). Design, synthesis and biological evaluation of a macrocyclic discodermolide/dictyostatin hybrid. Chem. Commun. (Camb.).

[123] Paterson I., Gardner N.M., Guzman E., Wright A.E. (2008). Total synthesis and biological evaluation of potent analogues of dictyostatin: Modification of the C2-C6 dienoate region. Bioorg. Med. Chem. Lett..

[124] Paterson I., Gardner N.M., Poullennec K.G., Wright A.E. (2007). Synthesis and biological evaluation of novel analogues of dictyostatin. Bioorg. Med. Chem. Lett..

[125] Paterson I., Gardner N.M., Poullennec K.G., Wright A.E. (2008). Synthesis and biological evaluation of 10,11-dihydrodictyostatin, a potent analogue of the marine anticancer agent dictyostatin. J. Nat. Prod..

[126] Shin Y., Fournier J.H., Bruckner A., Madiraju C., Balachandran R., Raccor B.S., Edler M.C., Hamel E., Sikorski R.P., Vogt A. (2007). Synthesis and biological evaluation of (−)-dictyostatin and stereoisomers. Tetrahedron.

[127] Shin Y., Fournier J.-H., Balachandran R., Madiraju C., Raccor B.S., Zhu G., Edler M.C., Hamel E., Day B.W., Curran D.P. (2005). Synthesis and biological evaluation of (−)-16-normethyldictyostatin: A potent analogue of (−)-dictyostatin. Org. Lett..

[128] Zampella A., D’Auria M.V., Minale L., Debitus C., Roussakis C. (1996). Callipeltoside A: A cytotoxic aminodeoxy sugar-containing macrolide of a new type from the marine Lithistida sponge *Callipelta* sp.. J. Am. Chem. Soc..

[129] Trost B.M., Gunzner J.L., Dirat O., Rhee Y.H. (2002). Callipeltoside A: Total synthesis, assignment of the absolute and relative configuration, and evaluation of synthetic analogues. J. Am. Chem. Soc..

[130] Evans D.A., Burch J.D., Hu E., Jaeschke G. (2008). Enantioselective total synthesis of callipeltoside A: Two approaches to the macrolactone fragment. Tetrahedron.

[131] Huang H., Panek J.S. (2004). Total synthesis of callipeltoside A. Org. Lett..

[132] Paterson I., Davies R.D.M., Heimann A.C., Marquez R., Meyer A. (2003). Stereocontrolled total synthesis of (−)-callipeltoside A. Org. Lett..

[133] Carpenter J., Northrup A.B., Chung D., Wiener J.J.M., Kim S., MacMillan D.W.C. (2008). Total synthesis and structural revision of callipeltoside C. Angew. Chem. Int. Ed..

[134] Zampella A., D’Auria M.V., Minale L., Debitus C. (1997). Callipeltosides B and C, two novel cytotoxic glycoside macrolides from a marine lithistida sponge *Callipelta* sp.. Tetrahedron.

[135] D’Auria M.V., Zampella A., Paloma L.G., Minale L., Debitus C., Roussakis C., le Bert V. (1996). Callipeltins B and C; bioactive peptides from a marine Lithistida sponge *Callipelta* sp.. Tetrahedron.

[136] Bassarello C., Zampella A., Monti M.C., Gomez-Paloma L., D’Auria M.V., Riccio R., Bifulco G. (2006). Quantum mechanical calculation of coupling constants in the configurational analysis of flexible systems: Determination of the configuration of callipeltin A. Eur J. Org. Chem..

[137] Zampella A., D’Orsi R., Sepe V., Casapullo A., Monti M.C., D’Auria M.V. (2005). Concise synthesis of all stereoisomers of β-methoxytyrosine and determination of the absolute configuration of the residue in callipeltin A. Org. Lett..

[138] Kikuchi M., Watanabe Y., Tanaka M., Akaji K., Konno H. (2011). Synthesis and cytotoxicity of the depsipeptides analogues of callipeltin B. Bioorg. Med. Chem. Lett..

[139] Allingham J.S., Zampella A., D’Auria M.V., Rayment I. (2005). Structures of microfilament destabilizing toxins bound to actin provide insight into toxin design and activity. Proc. Natl. Acad. Sci. USA.

[140] D’Auria M.V., Paloma L.G., Minale L., Zampella A., Verbist J.-F., Roussakis C., Debitus C., Patissou J. (1994). Reidispongiolide A and B, two new potent cytotoxic macrolides from the New Caledonian sponge *Reidispongia coerulea*. Tetrahedron.

[141] Paterson I., Ashton K., Britton R., Cecere G., Chouraqui G., Florence G.J., Knust H., Stafford J. (2008). Total synthesis of (−)-reidispongiolide A, an actin-targeting macrolide isolated from the marine sponge *Reidispongia coerulea*. Chem. Asian J..

[142] Fuwa H., Naito S., Goto T., Sasaki M. (2008). Total synthesis of (+)-neopeltolide. Angew. Chem. Int. Ed..

[143] Kartika R., Gruffi T.R., Taylor R.E. (2008). Concise enantioselective total synthesis of neopeltolide macrolactone highlighted by ether transfer. Org. Lett..

[144] Paterson I., Miller N.A. (2008). Total synthesis of the marine macrolide (+)-neopeltolide. Chem. Commun..

[145] Custar D.W., Zabawa T.P., Hines J., Crews C.M., Scheidt K.A. (2009). Total synthesis and structure activity investigation of the marine natural product neopeltolide. J. Am. Chem. Soc..

[146] Xie W., Ding D., Zi W., Li G., Ma D. (2008). Total synthesis and structure assignment of papuamide B, a potent marine cyclodepsipeptide with anti-HIV properties. Angew. Chem. Int. Ed..

[147] D’Auria M.V., Debitus C., Paloma L.G., Minale L., Zampella A. (2002). Superstolide A: A potent cytotoxic macrolide of a new type from the New Caledonian deep water marine sponge *Neosiphonia superstes*. J. Am. Chem. Soc..

[148] Tortosa M., Yakelis N.A., Roush W.R. (2008). Total synthesis of (+)-superstolide A. J. Org. Chem..

[149] Zhu J., Ma D. (2003). Total synthesis of microsclerodermin E. Angew. Chem. Int. Ed..

[150] Nakao Y., Oku N., Matsunaga S., Fusetani N. (1998). Cyclotheonamides E2 and E3, new potent serine protease inhibitors from the marine sponge of the genus *Theonella*. J. Nat. Prod..

[151] Fusetani N., Matsunaga S., Matsumoto H., Takebayashi Y. (1990). Bioactive marine metabolites. 33. Cyclotheonamides, potent thrombin inhibitors, from a marine sponge *Theonella* sp.. J. Am. Chem. Soc..

[152] Hagihara M., Schreiber S.L. (1992). Reassignment of stereochemistry and total synthesis of the thrombin inhibitor cyclotheonamide B. J. Am. Chem. Soc..

[153] Wipf P., Kim H. (1993). Total synthesis of cyclotheonamide A. J. Org. Chem..

[154] Wasserman H.H., Zhang R. (2002). Application of cyano ylide methodology to the synthesis of cyclotheonamides E2 and E3. Tetrahedron.

